# BHLHE40-mediated RGS16 upregulation: a driver propelling gastric cancer progression via ferroptosis suppression

**DOI:** 10.1186/s41065-025-00447-y

**Published:** 2025-05-24

**Authors:** Caiyun Guo, Hua Tang, Maifang Ren, Yongli Zhang

**Affiliations:** 1 Department of Gastroenterology, Xi’an International Medical Center Hospital, Xi’an, 710117 Shaanxi China; 2 Department of Gastroenterology, Tongchuan People’s Hospital, Tongchuan, 727100 Shaanxi China

**Keywords:** Gastric cancer, RGS16, BHLHE40, Ferroptosis

## Abstract

**Background:**

Gastric cancer (GC), a malignant neoplasm that arises from the epithelium of the gastric mucosa, endangers patients’ lives and health severely. Regulator of G-protein signaling 16 (RGS16) has been found to be correlated with the malignant progression of various cancers, and BHLHE40 is highly expressed in GC. However, it remains unclear whether there is a regulatory mechanism between the them.

**Methods:**

The bioinformatics tools were applied to assess the differentially expressed genes in GC. Next, the expression levels of mRNA and protein were evaluated by qRT-PCR and Western blot. Cellular behaviors were assessed using CCK-8, EdU, Transwell, and flow cytometry assays. Meanwhile, the ferroptosis-related indicators were measured. Subsequently, the xenograft models were set up to estimate the role of RGS16 in vivo. Besides, the interaction between BHLHE40 and RGS16 was determined using ChIP assay and dual-luciferase reporter assay.

**Results:**

RGS16 exhibited an upregulated pattern in GC. In addition, silencing RGS16 impeded the proliferation, migration and invasion of GC cells while reinforcing apoptosis and ferroptosis. Moreover, RGS16 boosted the growth of tumors in vivo. Furthermore, BHLHE40 could bind to RGS16 and positively regulate its expression. Overexpression of RGS16 reversed the effects of silencing BHLHE40 on GC cells.

**Conclusion:**

BHLHE40 curbed ferroptosis and oxidative stress of GC cells by modulating the expression of RGS16, thereby facilitating the malignant progression of GC.

**Supplementary Information:**

The online version contains supplementary material available at 10.1186/s41065-025-00447-y.

## Introduction

Gastric cancer (GC), a malignant tumor of the gastric epithelium, is a significant digestive tract malignancy [1]. With its incidence sharply rising after the age of 50 and peaking between 55 and 80 years old, GC has become a major health concern [2, 3]. The grim reality is reflected in its survival statistics: globally, the 5-year relative survival rate of GC patients lingers around 30–35%, and in advanced-stage cases, even in regions with advanced medical resources, the survival rate plummets below 20% [4]. This dismal situation not only exacts a heavy toll on patients’ lives but also burdens the global economy, underscoring the urgent need for breakthroughs in diagnostic and therapeutic approaches and a deeper exploration of the underlying molecular mechanisms driving GC development [5]. Current treatment modalities for GC, including traditional surgery, chemotherapy, and radiotherapy, are fraught with limitations. Surgical resection may not be feasible for advanced-stage patients, while chemotherapy and radiotherapy often cause severe side effects due to their lack of selectivity. Although targeted therapies and immunotherapies have emerged as promising alternatives, challenges such as drug resistance and high costs remain major obstacles [6]. These circumstances highlight the critical necessity of uncovering novel therapeutic targets and understanding the complex biological processes driving GC development.

Ferroptosis, a newly found cell death mode, varies from previously identified ones like apoptosis, autophagy, pyroptosis, and necrosis in terms of morphology, biochemistry, and the genes involved [7, 8]. It is now believed that ferroptosis restricts oncogene-driven cell proliferation and dedifferentiation, inhibits tumor growth and increases the sensitivity of a variety of tumors to chemotherapeutic agents and immunotherapy, and thus the induction of ferroptosis in cancer cells provides an important direction for expanding the ideas of tumor therapy [9–11]. Currently, multiple studies have indicated that ferroptosis plays an essential role in the pathogenesis and treatment of GC [12, 13]. A recent study demonstrated that, by leveraging OTUB1 to manage the stability of GPX4 protein, CST1 suppressed ferroptosis and simultaneously accelerated GC metastasis [14]. Moreover, emerging cell death pathways such as cuproptosis and disulfidptosis may also have potential associations with the behaviors of gastric cancer cells. Cuproptosis, a copper-dependent process, could affect copper metabolism and redox state in GC cells [15–17]. Disulfidptosis, triggered by disulfide stress on the actin cytoskeleton, might disrupt cell adhesion and cytoskeletal dynamics [18, 19]. These pathways may interact with ferroptosis, offering new targets for GC research.

The Regulator of G-protein signaling 6 (RGS16), a key member of the RGS family, functions as an intracellular negative regulator of G protein-coupled receptor (GPCR) signaling [20, 21]. During the last several years, it has been discovered that RGS16 participates in not only the regulation of cell growth and differentiation, material transport, cellular immune response and other physiological processes but also exerts an influence on the onset and progression of numerous tumors by regulating specific crucial proteins or growth factors [22, 23]. RGS16 has drawn increasing attention for its significant role in malignancies. It has been documented that RGS16 modulated Hippo-YAP activity to fuel esophageal cancer cell proliferation and migration. Meanwhile, it also propelled glioma progression and functions as a prognostic factor [24, 25], which together underline its far-reaching influence on tumor progression and clinical prognosis, thus making it an essential target worthy of in-depth exploration across diverse cancer types. Nevertheless, despite its well-established roles in various cancers, the function of RGS16 in GC, especially its involvement in ferroptosis regulation, remains largely unknown.

On the other hand, BHLHE40, a transcription factor belonging to the basic helix-loop-helix (bHLH) family, has been implicated in multiple cellular processes, such as cell proliferation, differentiation, and development [26]. In cancer biology, BHLHE40 has been reported to promote colon cancer cell growth and migration by upregulating INHBA [27]. In GC, it is activated by HIF-1α under hypoxic conditions, facilitating tumor progression [28]. Moreover, the upregulation of BHLHE40 expression has been correlated with poor differentiation of GC, suggesting its crucial role in tumor development [29]. So far, whether BHLHE40 can regulate the RGS16 gene and thereby influence ferroptosis and tumorigenesis in GC cells has not been explored.

Against this backdrop, the present study aims to investigate the roles of RGS16 and BHLHE40 in GC progression, with a particular focus on elucidating the regulatory relationship between these two molecules and their impact on ferroptosis. By exploring these uncharted territories, we hope to provide novel insights into the molecular mechanisms of GC, identify potential therapeutic targets, and ultimately contribute to the development of more effective treatment strategies for GC patients.

## Materials and methods

### Clinical samples

Thirty-five pairs of primary GC tissues and their matching adjacent non-cancerous tissues were gathered from patients who underwent gastric resection due to GC at the Xi’an International Medical Center Hospital. It was worth noting that none of these participants had undergone preoperative chemotherapy or radiotherapy prior to enrollment. All patients willingly signed written informed consent forms, allowing this study to progress in complete alignment with the principles laid out by the Declaration of Helsinki. Approval for this study was granted by the Ethics Committee of the Xi’an International Medical Center Hospital (Human ethics number: 2023029).

### Cell lines

Normal human gastric mucosal epithelial cell line (GES-1) and GC cell lines (NCI-N87 and HGC-27) were purchased from the Suncell Biotechnology Co., Ltd. (Wuhan, China), which were cultured in RPMI-1640 medium containing 10% fetal bovine serum (FBS) (Thermo Fisher Scientific, Rockville, MD, USA) and 1% penicillin/streptomycin (P/S) (Invitrogen, Carlsbad, CA, USA). The HEK-293T cells were procured from the Princella Life Technology Co., Ltd. (Wuhan, China) and cultured in Dulbecco’s modified Eagle’s medium DMEM (Invitrogen) supplemented with 10% FBS (Thermo Fisher Scientific) and 1% P/S (Invitrogen). The culturing was carried out under standard cell culture conditions, precisely in a humidified incubator maintaining an environment of 5% CO_2_ and 95% air at a steady temperature of 37℃, to ensure the optimal growth and viability of the cells.

### Bioinformatics analysis

The GEO database, a public repository storing a vast amount of gene expression and other genomic data for global research use (https://www.ncbi.nlm.nih.gov/geo/geo2r/?acc=GSE79973), was employed to analyze the genes that were upregulated or downregulated in normal tissue samples and tumor samples. Genes with a fold-change of at least 1.5 and a *P*-value less than 0.05 were considered significantly differentially expressed. UALCAN, an online tool for analyzing and visualizing cancer genomic data, was applied to show the expression of RGS16 in Stomach Adenocarcinoma (STAD) based on TCGA samples (https://ualcan.path.uab.edu/cgi-bin/TCGAExResultNew2.pl?genenam=RGS16&ctype=STAD). GEPIA, an online tool for gene expression and survival analysis in cancers, was employed to assess the expression of RGS16 in different diseases (http://gepia.cancer-pku.cn/detail.php?gene=RGS16). Furthermore, the hTFtarget (https://guolab.wchscu.cn/hTFtarget/#!/tf) was utilized to predict the potential transcription factors that may regulate RGS16. The correlation between the expression levels of RGS16 and BHLHE40 was evaluated and presented by the TIMER database (https://cistrome.shinyapps.io/timer/). Finally, the Jasper website (https://jaspar.elixir.n) was utilized for the prediction of binding sites between RGS16 and BHLHE40.

### Quantitative real-time polymerase chain reaction (qRT-PCR)

To verify the results of bioinformatics, qRT-PCR assay was performed to measure RGS16 mRNA levels in clinical samples and GC cell lines. TriQuick Reagent (Solarbio, Beijing, China) was utilized to isolate the total RNA from tissues and cells. Then, the Transcriptor First Strand cDNA Synthesis Kit (Roche, Vilvoord, Brussel, Belgium) was used to obtain the cDNA. Subsequently, the qRT-PCR was performed using SYBR Green Realtime PCR Master Mix (Toyobo Co., Osaka, Japan) with Real-Time PCR Detection System (Bio-Rad, Shanghai, China). The primers are as follows: RGS16: Forward (5’-3’): CAAGTTCGAGTGGGGCAGTA, Reverse (5’-3’): CTTTAGGGGCCTCACTGCAA. BHLHE40: Forward (5’-3’): TGCCACGTAGGAATTGTTTGT, Reverse (5’-3’): ACGGTTCCCATCCTACCAGA. β-actin: Forward (5’-3’): GGATTCCTATGTGGGCGACGA, Reverse (5’-3’): GCGTACAGGGATAGCACAGC.

### Western blot analysis

Proteins were isolated from tissues and cells with the aid of the RIPA Lysis and Extraction Buffer (Thermo Fisher Scientific). Upon separation of the protein samples via SDS-PAGE, they were transferred onto PVDF membranes (Millipore, Billerica, MA, USA) for further analysis. Firstly, the membrane was blocked with 5% non-fat milk at 4℃ overnight. After that, the membrane was respectively incubated with the primary antibodies and then the secondary antibodies at room temperature (RT) for 1 h. After that, the membrane was developed using the BeyoECL Plus Kit (Beyotime). Finally, the membrane was imaged and analyzed using a gel imaging system. The primary antibodies include Anti-RGS16 (1:500, Cat: ab119424, Abcam, Cambridge, UK), Anti-SHARP2/DEC1 (1:500, Cat: ab259837, Abcam), Anti-Glutathione Peroxidase 4 (1:500, Cat: ab125066, Abcam) and Anti-beta Actin (1:500, Cat: ab8226, Abcam). The secondary antibodies include Goat Anti-Rabbit IgG H&L (HRP) (1:5000, Cat: ab6721, Abcam) and Goat Anti-Mouse IgG H&L (HRP) (1:5000, Cat: ab6789, Abcam).

### Cell transfection

GeneChem (Shanghai, China) was responsible for the design of short hairpin RNAs (shRNAs) that target RGS16 (sh-RGS16), BRD3 (sh-BRD3),CTCF (sh-CTCF) and BHLHE40 (sh-BHLHE40). In this experiment, nontargeted shRNAs were used as negative controls, labeled as sh-NC. To obtain the RGS16-overexpression plasmids (oe-RGS16), the full-length RGS16 sequence was inserted into pcDNA3.1 plasmids provided by GenePharma (Shanghai, China). For the transfection process, the Lipofectamine 2000 (Invitrogen) was employed following the manufacturer’s instructions. The final concentration of shRNAs used for transfection was 50 nM, and the concentration of plasmids (including oe-RGS16 and control plasmids) was 1 µg per well in 6-well plates. These concentrations were optimized in preliminary experiments to ensure efficient transfection and minimal cytotoxicity while achieving significant gene knockdown or overexpression effects.

### Cell viability assay

The Cell Counting Kit-8 (Beyotime) was adopted to detect the viability of GC cells under different conditions. All experimental procedures strictly followed the instructions in the manual. Cells were seeded into a 96-well plate at a density of 2 × 10^3^ cells/well. After 24 h, 10 µL of CCK-8 solution was added into each well. Then, the cell plate was placed into a 37℃ incubator for 2 h. Eventually, the absorbance at 450 nm was measured using a Microplate Reader.

### Cell proliferation assay

The BeyoClick™ EdU Cell Proliferation Kit with Alexa Fluor 594 was utilized to evaluate the proliferative ability of GC cells. To begin with, the NCI-N87 and HGC-27 cells were seeded into a 6-well plate and cultured at 37℃ for 24 hours. An equal volume of 2×EdU solution (20 µM) to that of the medium in the plate was introduced into each well to make the final concentration 10 µM, and then, the incubation was continued for 2 hours. Subsequently, the cells were fixed and permeabilized successively by 4% paraformaldehyde (Beyotime) and 0.3% TritonX-100 (Beyotime). Then, the cells were counterstained with 4’,6-diamidino-2-phenylindole (DAPI) (Thermo Fisher Scientific). A fluorescence microscope was used to observe the staining of the cells.

### Transwell assay

Transwell assay was carried out utilizing the Transwell chambers (8-µm pore size; Corning, Tewksbury, MA, USA) to estimate the cell migration and invasion [30]. Upon trypsin digestion, a suspension of 1 × 10^5^ cells, which had been re-suspended in 200 µL of serum-free medium, was inoculated into the upper chamber. Simultaneously, 500 µL of complete medium supplemented with 10% FBS was added to fill the lower chamber of the Transwell plate. After a 24-hour incubation period, the cells remaining on the surface of the upper chamber were carefully wiped off with a cotton swab. Regarding invasion detection, 60 µL of Matrigel matrix (diluted by serum-free medium) (Bedford, MA, USA) was used to pre-coat the bottom of the upper chamber before the cells were seeded. The remaining steps were identical to those described above. The cells were first fixed using methanol and then stained with the crystal violet solution. Subsequently, the migrated and invade cells were counted under a microscope.

### Detection of cell apoptosis by flow cytometry

Flow cytometry was employed for the detection of cell apoptosis with the utilization of Annexin V-FITC Cell Apoptosis Detection Kits (Beyotime). In brief, after digestion and counting of the cells, 5 × 10^4^ cells were resuspended in 195 µL of Annexin V-FITC binding buffer. Subsequently, 5 µL of Annexin V-FITC was added to the cells and gently mixed. Then, 10 µL of propidium iodide (PI) was added. Following a 10-minute incubation at RT in the dark, flow cytometry was utilized to analyze the apoptosis of the cells.

### Evaluation of oxidative stress and ferroptosis

The intracellular ROS level was detected using Reactive Oxygen Species (ROS) Fluorometric Assay Kit (Yeasen, Shanghai, China) as the protocol. For the detection of Malondialdehyde (MDA), Glutathione (GSH), and Fe^2+^ levels in GC cells, the Lipid Peroxidation MDA Assay Kit (Beyotime), the GSH and GSSG Assay Kit (Beyotime), and the Ferrous Ion Content Detection Kit (Solarbio) were respectively utilized according to the instructions of the kits. Furthermore, the amount of GPX4 protein was analyzed by Western blot.

### Establishment of xenograft tumor model

6–8 weeks female BALB/c nude mice were procured from Aniphe Biotechnology Co., Ltd. (Jiangsu, China) and housed in a suitable environment in line with the breeding requirements detailed on the website. The mice were grouped based on the injected cell types, namely sh-NC and sh-RGS16, with five mice in each group. To establish a subcutaneous transplantation model of GC, 100 µL of NCI-N87 cells at a concentration of 10^6^ cells/µL were subcutaneously injected into the right armpit of mice. Following this, tumor volume was documented at an interval of five days (volume (mm³) = width² × length/2). After 25 days of subcutaneous injection, the mice in each group were sacrificed and the tumor tissues were excised, weighed, and photographed. All the animal experiments were conducted in accordance with the protocols endorsed by the Ethics Committee on Pre-Clinical Studies of Xi’an International Medical Center Hospital, aligning with the established International Guiding Principles for Animal Research (Animal ethics number: 2024-0015).

### Immunohistochemistry (IHC)

The tumor tissues collected from the preceding experiment were fixed in 10% formalin for 12 hours. Thereafter, the tissues were treated with ethanol and xylene and then embedded in paraffin. After that, the tissues were sliced into 4 micron-thick slices. Following antigen retrieval (high-temperature and high-pressure antigen retrieval method), the tissue slices were prepared for subsequent immunological reactions. The slides were incubated with the primary antibodies including anti-Ki67 (Cat: ab15580, Abcam), anti-GXP4 (Cat: ab75810, Abcam), and anti-RGS16 (Cat: ab119424, Abcam) at RT for 1 hour. After being washed using PBS for three times, the HRP-conjugated secondary antibodies Goat Anti-Rabbit/Mouse IgG (ab6721/ab6728, Abcam) were incubated with the slides following the same procedures as before. For visualization purposes, 3,3’-diaminobenzidine (DAB) was employed as the chromogenic substrate for counterstaining. Finally, the staining patterns of the tissues were observed and recorded under a microscope.

### Chromatin immunoprecipitation (ChIP) assay

To identify the enrichment degree of RGS16 promoter, a ChIP experiment was carried out. Firstly, the 1% formaldehyde solution was used to cross-link DNA-protein complexes. After being kept at 37℃ for 10 min, 0.125 M glycine was added for 5-minute incubation to stop the cross-linking reaction. Thereafter, chromatin was released by lysing the cells with a lysis buffer (containing protease inhibitors and RNase inhibitors). Subsequently, the cell suspension was placed on ice and sonicated to fragment the chromatin. For IP assay, the BHLHE40 antibody (Cat: ab70723, Abcam) and IgG antibody (Cat: ab172730, Abcam) (used as control) were added to pre-washed Protein A/G magnetic beads (Thermo Fisher Scientific) and incubated at 4℃ for 4 h. Following this, the chromatin fragment solution was incubated with the antibody-magnetic bead complex overnight at 4℃. Non-specifically bound chromatin fragments were removed by washing the magnetic beads multiple times. Protease K (Yeasen) was added to the magnetic bead-antibody-chromatin complex and incubated at 65℃ for 4 h to reverse the cross-linking. After DNA purification, qPCR was performed. By comparing the Ct values of the target genes (Site 1 and Site 2) with those of the reference gene (β-actin), the relative enrichment amounts at Site 1 and Site 2 were calculated.

### Dual-luciferase reporter assay

The HEK-293T cells were introduced with sh-NC and sh-BHLHE40 before this assay. The wild-type sequence (containing the normal RGS16 gene promoter region) and the mutant-type sequence (with specific mutations in the RGS16 gene promoter region) were cloned into pGL3-basic vector (Promega, Madison, WI, USA) for the construction of pGL3-basic-RGS16-WT/MUT. With the application of the polyfect transfection reagent (Qiagen, Germantown, MD, USA), the HEK-293T cells were simultaneously transfected with the constructed vectors and the pRL-TK vector, a renilla luciferase reporter plasmid acting as an internal control. Following 48 h of transfection, the cells were collected and lysed. Subsequently, the dual luciferase reporter assay kit (Promega) was applied to perform the dual-luciferase assay.

### Statistical analysis

Graphpad Prism 8.0.1 software was utilized for all statistical analyses. All experiments were independently replicated over three times, with the data presented as mean ± standard deviation (SD). The statistical analysis of disparities among two or multiple groups was conducted by Student’s *t* test and One-way and two-way ANOVA. The Pearson correlation coefficient was employed to determine the strength and direction of the linear relationship between the expression levels of RGS16 and BHLHE40. Statistical significance was considered at *P* < 0.05.

## Results

### The expression of RGS16 was upregulated in GC

**Fig. 1:**
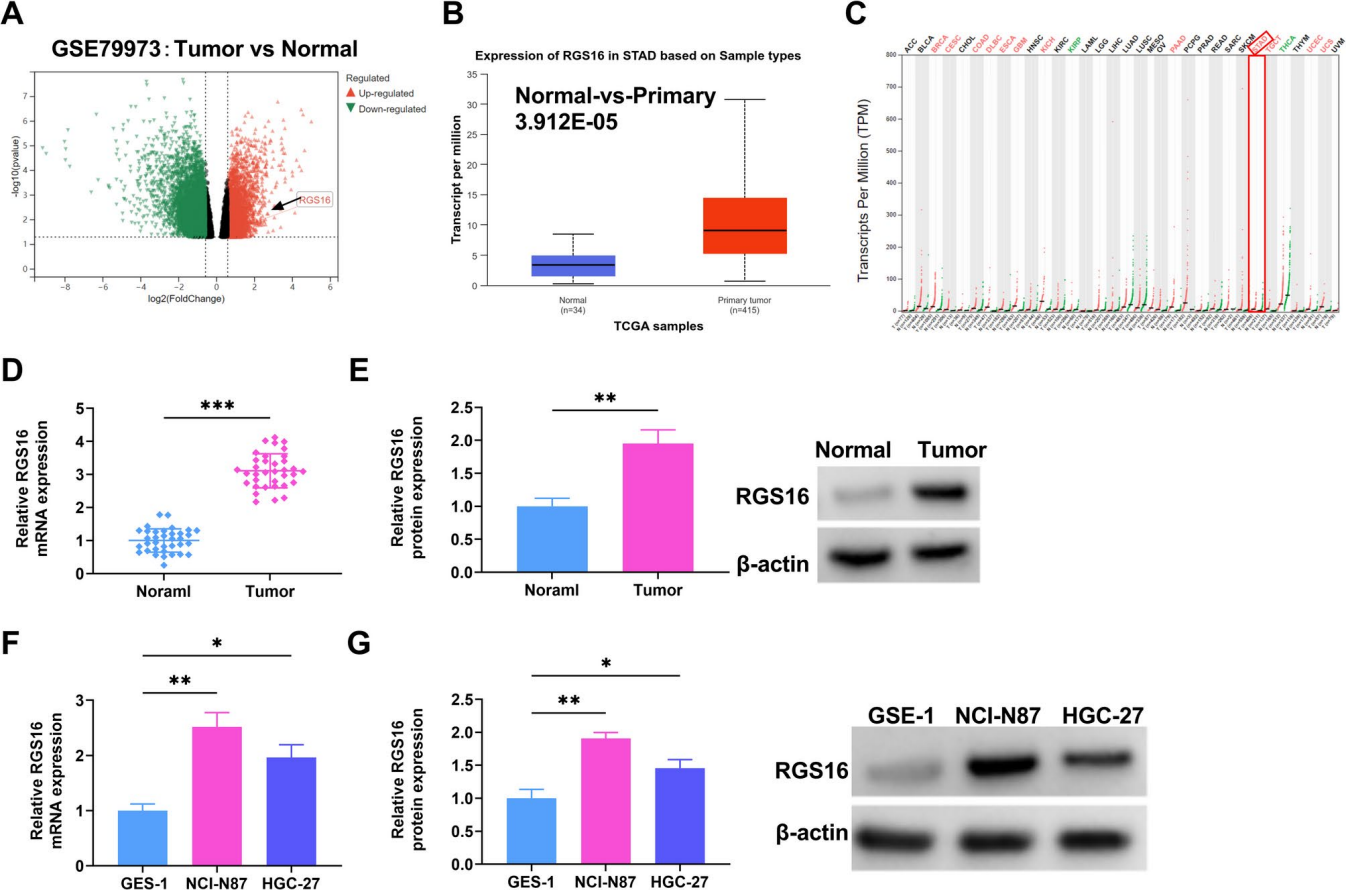
RGS16 is elevated in GC. (**A**) The expression levels of differentially expressed genes in GC and Control samples according to RNA-seq data from GEO dataset (GSE79973) were displayed. (**B**) UALCAN databases showed RGS16 expression in STAD based on the Cancer Genome Atlas (TCGA) samples (Normal: *n* = 34, Primary tumor: *n* = 415). (**C**) The GEPIA database showed the upregulation and downregulation of RGS16 expression in multiple diseases. (**D**) qRT-PCR assay was applied to measure the expression level of RGS16 mRNA in 35 pairs of GC tumor tissues and adjacent normal tissues. (**E**) Western blot analysis of RGS16 protein expression level in normal and tumor tissues. (**F**-**G**) The RGS16 mRNA and protein levels in GSE1, NCI-N87, and HGC-27 cells were detected by qRT-PCR and Western blot. * *P* < 0.05, ** *P* < 0.01, *** *P* < 0.001

Using the GEO database, the genes upregulated in GC were screened (GSE79973), and it was found that the RGS16 gene was upregulated in tumor tissues (Fig. 1A). The findings from the UALCAN database indicated that RGS16 expression in 415 primary tumor samples was markedly higher, approximately 3-fold compared to that in 34 normal samples (Fig. 1B). Subsequently, the GEPIA database was employed to display the upregulation and downregulation status of RGS16 in different diseases. Obviously, RGS16 exhibited an increased expression in STAD (Fig. 1C). To validate the results of bioinformatics, qRT-PCR assay was carried out on 35 pairs of GC and normal tissues, indicating that RGS16 mRNA showed elevated expression in GC tissues (Fig. 1D). Additionally, the results of Western blot analysis revealed that the expression of RGS16 protein was greater in GC tissues than normal tissues (Fig. 1E). Similarly, the expressions of RGS16 mRNA and protein in GC cells (NCI-N87 and HGC-27) were significantly higher than those in GES-1 (Fig. 1F-G). Collectively, RGS16 exhibited high-level expression in GC tissues and cells.

### Malignant proliferation and invasion of GC cells was suppressed by knockdown of RGS16

**Fig. 2:**
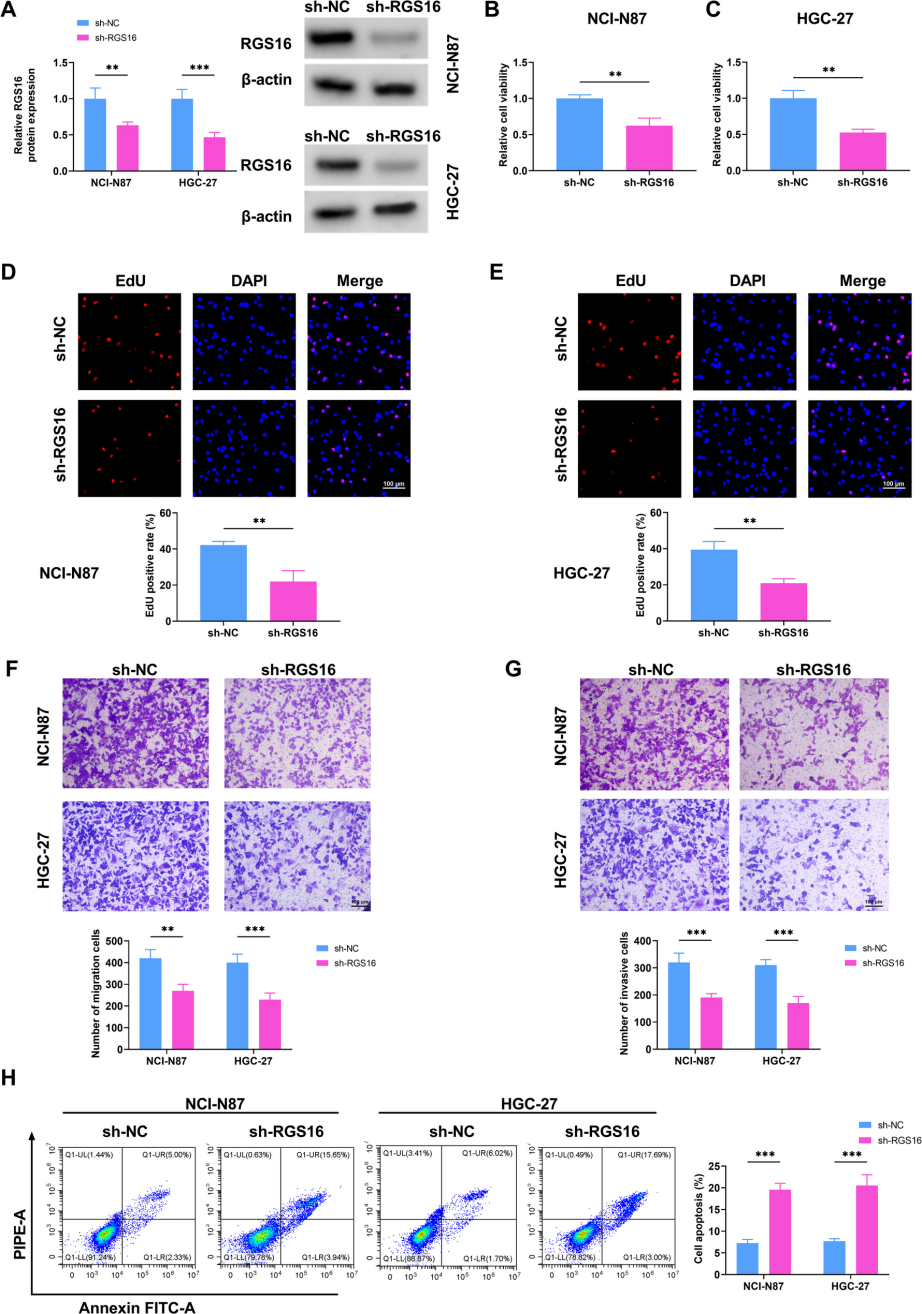
Malignant proliferation and invasion of GC cells can be suppressed by knockdown of RGS16. NCI-N87 and HGC-27 cells were transfected with sh-NC and sh-RGS16. (**A**) Western blot was utilized to verify the transfection efficiency. (**B**-**C**) Cell viability was determined by CCK-8 assay. (**D**-**E**) EdU assay was used to evaluate proliferation of GC cells. (**F**-**G**) Transwell assay was performed to assess the migration and invasion of GC cells transfected with sh-NC and sh-RGS16. (**H**) Cell apoptosis was detected by flow cytometry. ** *P* < 0.01, *** *P* < 0.001

The GC cell lines (NCI-N87 and HGC-27) were transfected with sh-RGS16, along with sh-NC as the negative control. In Western blot analysis, it was observed that the level of RGS16 in the sh-RGS16 group was lower compared to that in the sh-NC group (Fig. 2A). Then, the CCK-8 assay illustrated that cell viability decreased with the downregulation of RGS16 (Fig. 2B-C). Likewise, GC cells treated with sh-RGS16 showed inhibited cell proliferation (Fig. 2D-E). Next, cell migration and invasion analysis utilizing Transwell assay suggested that RGS16 silencing markedly restrained the migration and invasion of GC cells (Fig. 2F-G). Conversely, cell apoptosis was accelerated by the downregulation of RGS16 (Fig. 2H). To sum up, the downregulation of RGS16 curbed the viability, proliferation, migration as well as invasion of GC cells, while simultaneously promoted their apoptosis.

### Knockdown of RGS16 facilitated ferroptosis and oxidative stress of GC cells

**Fig. 3:**
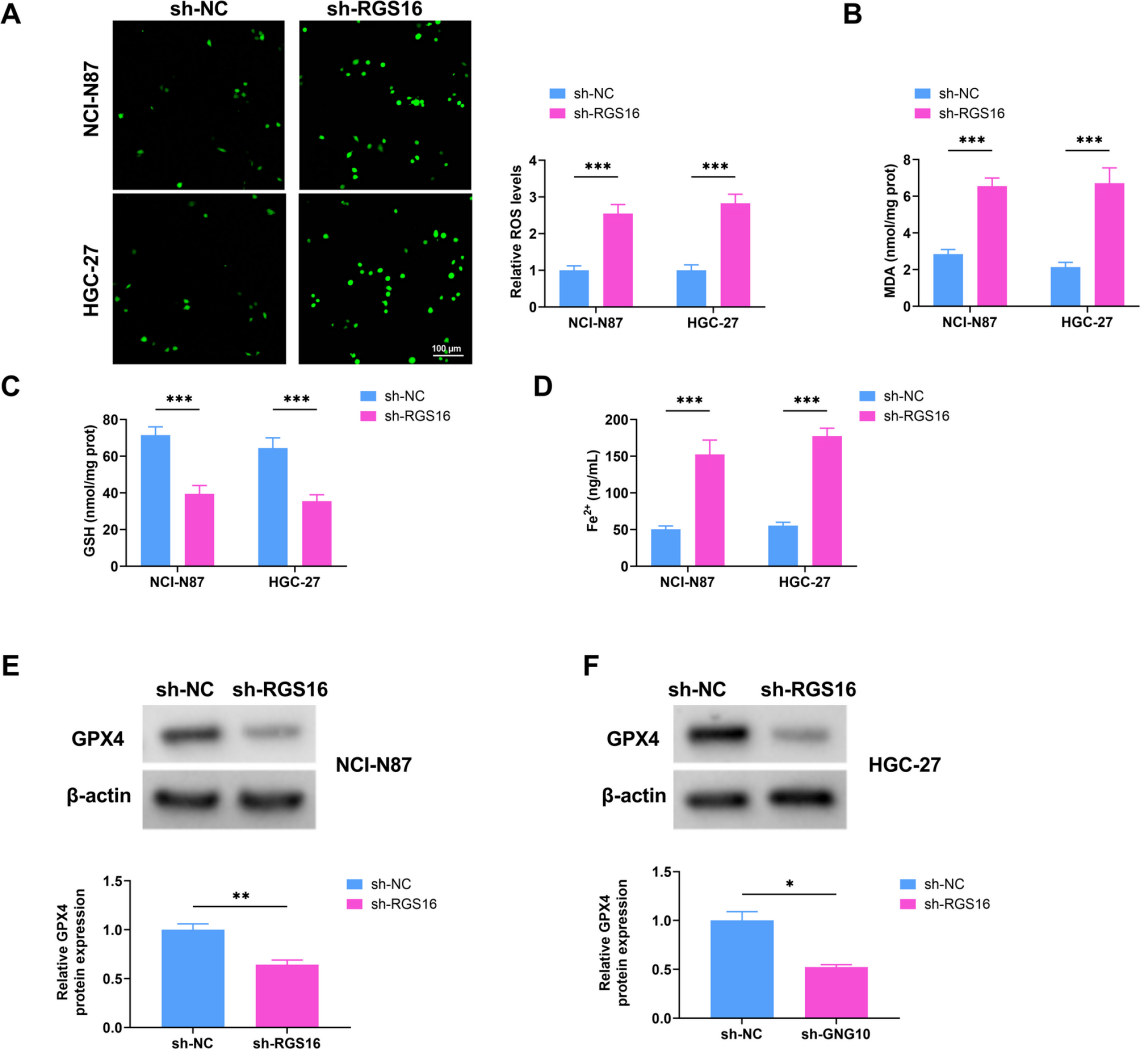
Silencing RGS16 increases ferroptosis and oxidative stress of GC cells. (**A**) The ROS levels of NCI-N87 and HGC-27 cells transfected with sh-NC and sh-RGS16 were measured by using corresponding kits. (**B**-**D**) The contents of MDA, GSH, and Fe^2+^ were detected using commercial kits. (**E**-**F**) Western blot analysis of GPX4 protein levels in NCI-N87 and HGC-27 cells. * *P* < 0.05, ** *P* < 0.01, *** *P* < 0.001

Some indicators were detected by using commercial kits to further validate the function of RGS16 in ferroptosis and oxidative stress of GC cells. First, the ROS levels in GC cells were assessed by fluorescence microscopy. As depicted in Fig. 3A, the intracellular ROS level was elevated in cells treated with sh-RGS16 relative to the control (sh-NC). Besides, the content of MDA was conspicuously elevated after GC cells were transfected with sh-RGS16 (Fig. 3B). In addition, the GSH level in NCI-N87 and HGC-27 cells decreased with the knockdown of RGS16, while the Fe^2+^ concentration significantly increased after RGS16 downregulation (Fig. 3C-D). Moreover, the GPX4 protein levels were tasted by Western blot. RGS16 silencing effectively retarded the expression of GPX4 protein (Fig. 3E-F). The above experimental data confirmed that the downregulation of RGS16 contributed to ferroptosis and oxidative stress of GC cells.

### Downregulation of RGS16 constrained tumor growth of GC in vivo

**Fig. 4:**
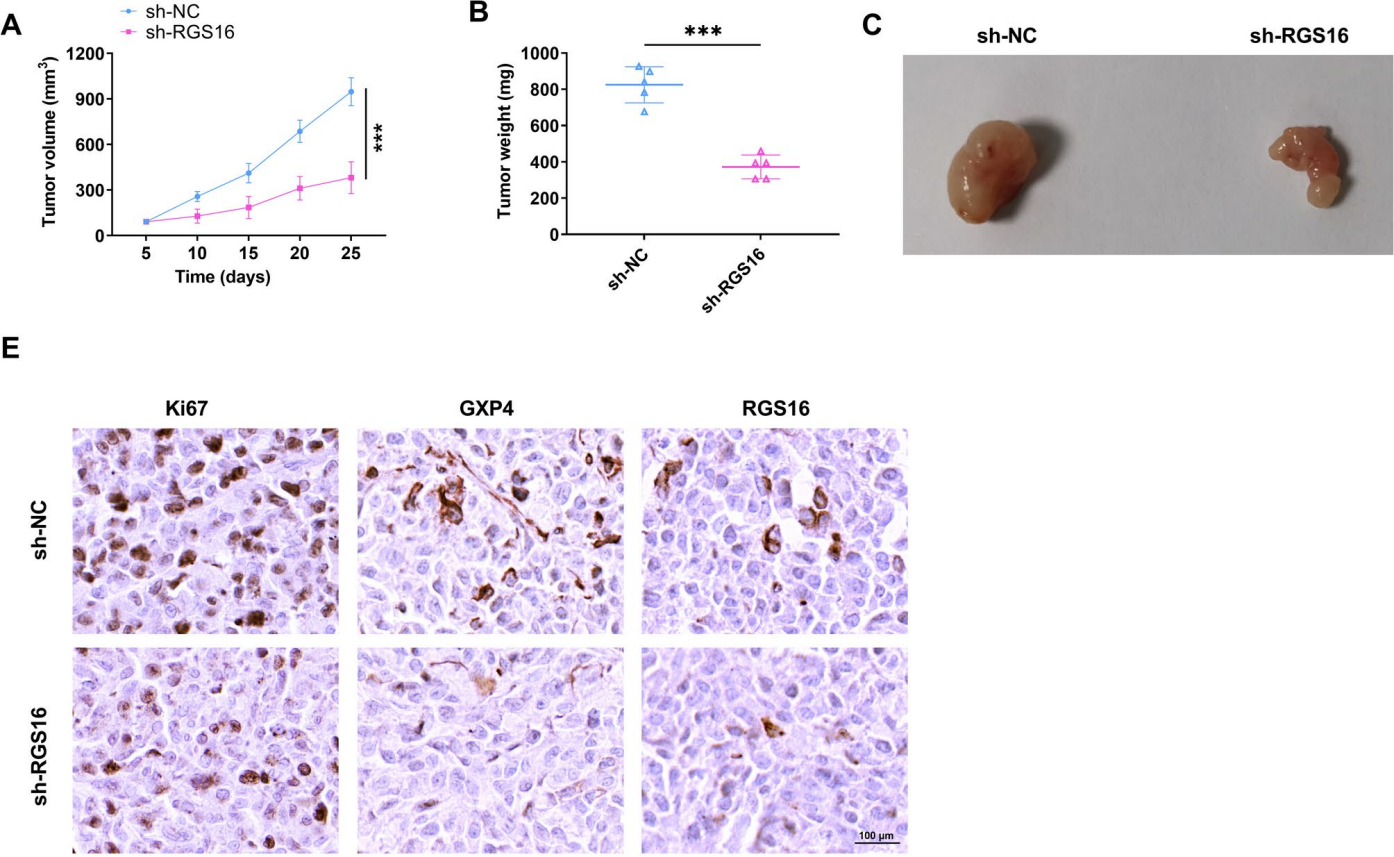
Knockdown of RGS16 suppresses tumor growth of GC in vivo. Tumors were harvested from the xenograft mouse models treated with sh-NC and sh-RGS16. (**A**) Tumor growth curves, (**B**) tumor weight and (**C**) presentative images. (**D**) IHC staining of Ki67, GXP4, and RGS16 from the xenograft tumors. *** *P* < 0.001

Further animal experiments were carried out to elucidate the functional role of RGS16 in tumor growth. By recording the changes in tumor volume within 25 days, it was observed that the tumor volume expansion of mice subject to sh-RGS16 treatment was decelerated in contrast to that of mice having received sh-NC treatment (Fig. 4A). The tumor tissues were isolated, weighed, and photographed. As shown in Fig. 4B-C, after RGS16 knockdown, the tumor weights and sizes were lower than those of the control group. Ki67 is widely used as a marker for assessing the proliferation of cancer cells [31]. IHC staining showed that after RGS16 was downregulated, the expression levels of Ki67 and RGS16 showed a decline, and the GPX4 protein in tumor tissues was also reduced (Fig. 4D). Together, these results demonstrated that the downregulation of RGS16 hindered the tumor growth of GC in vivo.

### BHLHE40 was positively related with RGS16 in GC

**Fig. 5:**
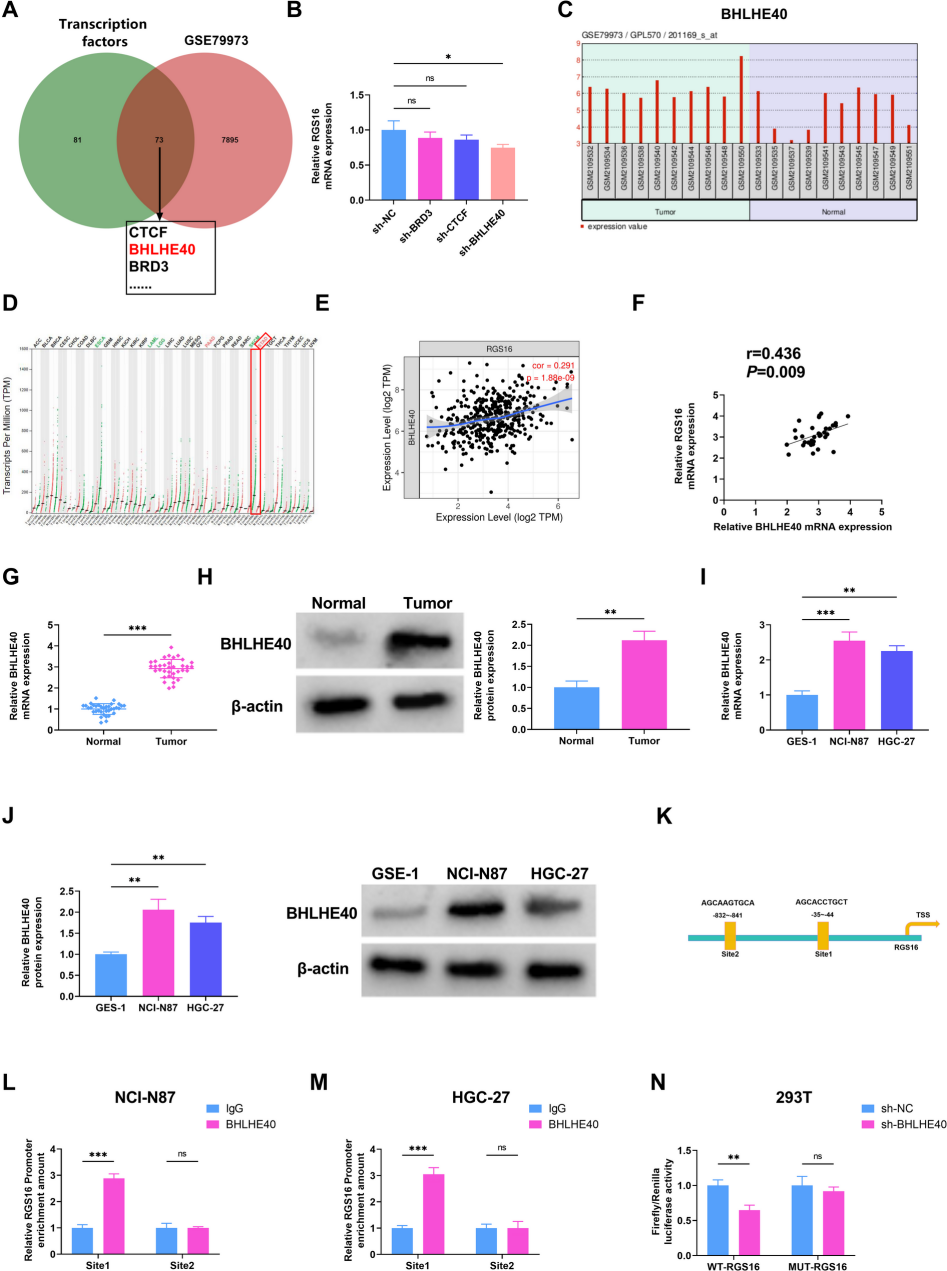
The interaction between BHLHE40 and RGS16. (**A**) Venn diagram depicting the intersection genes predicted by the GES79973 dataset and the hTFtarget website. (**B**) qRT-PCR was employed to test the PRG16 mRNA levels in cells treated with sh-NC, sh-BRD3, sh-CTCF, and sh-BHLHE40. (**C**) The GEO database was utilized to display the differential expression of BHLHE40 in tumor and normal tissues. (**D**) The upregulation or downregulation of BHLHE40 expression in different diseases was demonstrated by GEPIA database. (**E-F**) The TIMER database and qRT-PCR showed that the expressions of RGS16 and BHLHE40 were positively correlated. (**G**) qRT-PCR analysis of BHLHE40 mRNA levels in 35 pairs of normal and tumor tissues. (**H**) The BHLHE40 protein levels in normal and tumor tissues were examined by Western blot. (**I-J**) qRT-PCR and Western blot were used to detect the mRNA and protein levels of BHLHE40 in normal gastric epithelial cell (GES-1) and GC cells (NCI-N87 and HGC-27). (**K**) The Jasper database was utilized to show the potential binding sites of BHLHE40 and RGS16 promoter (Site 1:35–44 bp, Site 2: 832–841 bp). (**L-M**) The degree of BHLHE40 at the two binding sites of RGS16 was confirmed by ChIP assay. (**N**) Dual luciferase reporter assay was used to determine the effect of BHLHE40 knockdown on the luciferase activity of WT-RGS16 and MUT-RGS16 in 293T cells. * *P* < 0.05, ** *P* < 0.01, *** *P* < 0.001

Accordingly, the regulatory factors that interact with RGS16 were further investigated. By utilizing the hTFtarget website and GSE79973, 73 transcription factors with potential binding capabilities to RGS16 were screened out in GC (Fig. 5A). For further screening, the top three genes were subjected to knockdown procedures. The qRT-PCR results revealed that, in contrast to BRD3 and CTCF, RGS16 mRNA level was dramatically reduced following the downregulation of BHLHE40 (Fig. 5B). Furthermore, the GEO and GEPIA databases demonstrated that BHLHE40 expression showed an increase in GC (Fig. 5C-D). Based on the TIMER database and qRT-PCR, BHLHE40 and RGS16 were positively correlated in terms of expression levels (Fig. 5E-F). Additionally, the results of qRT-PCR and Western blot verified that both the BHLHE40 mRNA and protein were elevated in GC tissues and cells compared to those in normal counterparts and cells (Fig. 5G-J). Through the prediction by the Jasper website, two potential binding sites were discovered, namely site 1 (35–45 bp) and site 2 (832–842 bp) (Fig. 5K). Moreover, the ChIP assay was carried out to identify the enrichment level of RGS promoter using BHLHE40 antibody. The results validated that RGS16 had a relatively high enrichment level at site 1 (Fig. 5L-M). Knocking down BHLHE40 significantly suppressed the luciferase activity of wild-type RGS16. However, in the case of mutant RGS16, the knockdown of BHLHE40 had little influence on its luciferase activity (Fig. 5N). In conclusion, BHLHE40 could bind to the promoter of RGS16 and their expression levels showed a positive correlation.

### RGS16 overexpression reversed the effects of sh-BHLHE40 on GC cells regarding proliferation, migration, invasion and apoptosis

**Fig. 6:**
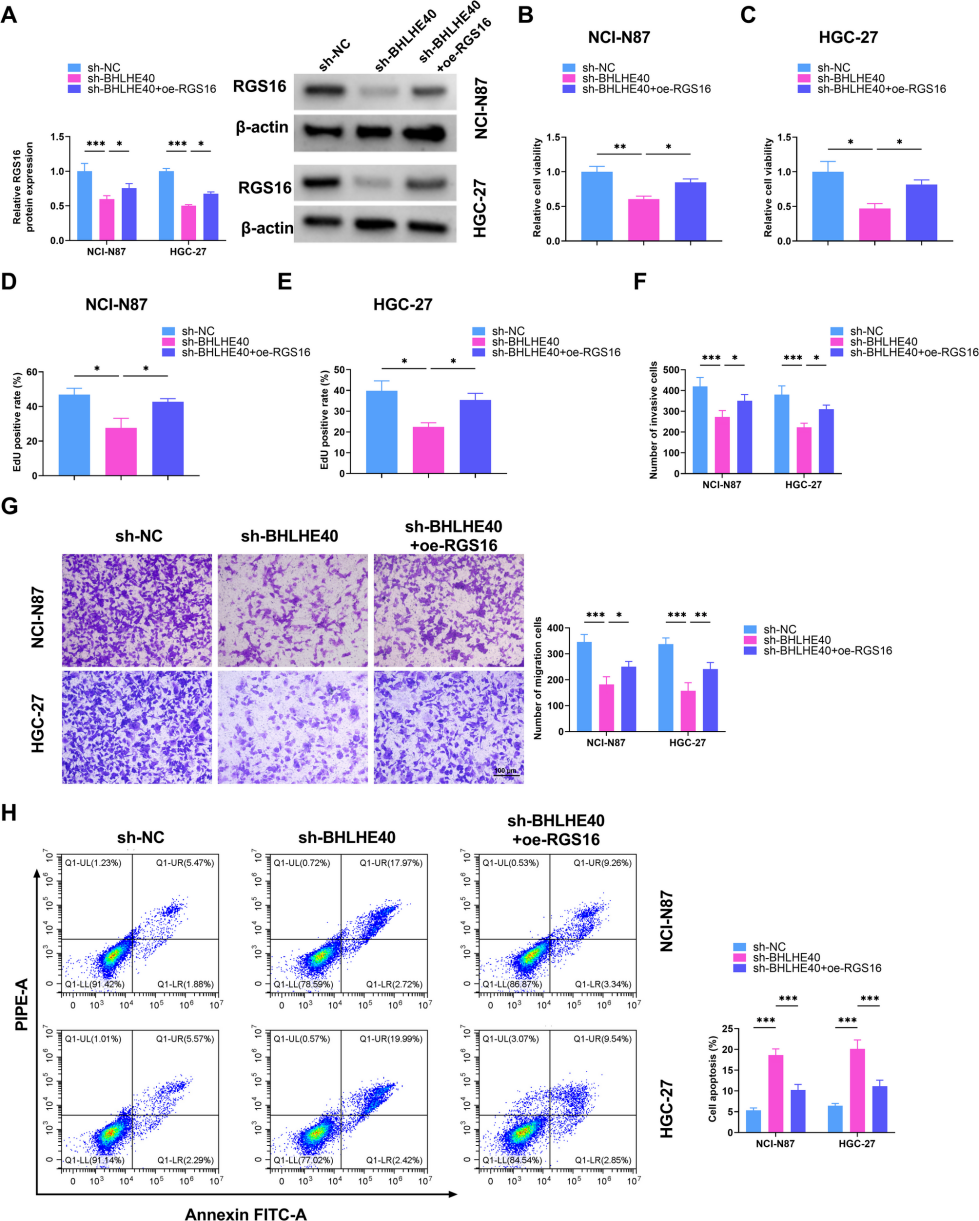
Overexpression of RGS16 reverses the inhibitory effects of BHLHE40 downregulation on cell malignant behaviors. NCI-N87 and HGC-27 cells were divided into 3 groups: sh-NC, sh-BHLHE40, and sh-BHLHE40 + oe-RGS16. (**A**) Western blot analysis of RGS16 expression in NCI-N87 and HGC-27 cells. (**B**-**C**) Cell viability of GC cells transfected with sh-NC, sh-BHLHE40, and sh-BHLHE40 + oe-RGS16. (**D**-**G**) Cell proliferation, invasion, and migration detected by EdU and Transwell assays in GC cell lines. (**H**) Flow cytometry for cell apoptosis. * *P* < 0.05, ** *P* < 0.01, *** *P* < 0.001

Using Western blot analysis, RGS16 expression in GC cells transfected with sh-NC, sh-BHLHE40, and sh-BHLHE40 + oe-RGS16 was detected. The RGS16 expression level was markedly decreased in the sh-BHLHE40 group compared to that in the sh-NC group, while it was increased in the sh-BHLHE40 + oe-RGS16 group, indicating that BHLHE40 regulated RGS16 expression (Fig. 6A). Likewise, the suppression effect of cell viability, proliferation, migration, and invasion due to BHLHE40 silencing was mitigated with the upregulation of RGS16 (Fig. 6B-G). Consistently, the intensified cell apoptosis due to BHLHE40 knockdown was reverted due to the overexpression of RGS16 (Fig. 6H). In summary, the repressive influences on cell viability, proliferation, migration, and invasion, and the promotive effect on cell apoptosis owing to the downregulation of BHLHE40 were counteracted by the overexpression of RGS16.

### The overexpression of RGS16 abolished the effect of BHLHE40 silencing on ferroptosis in GC cells

**Fig. 7:**
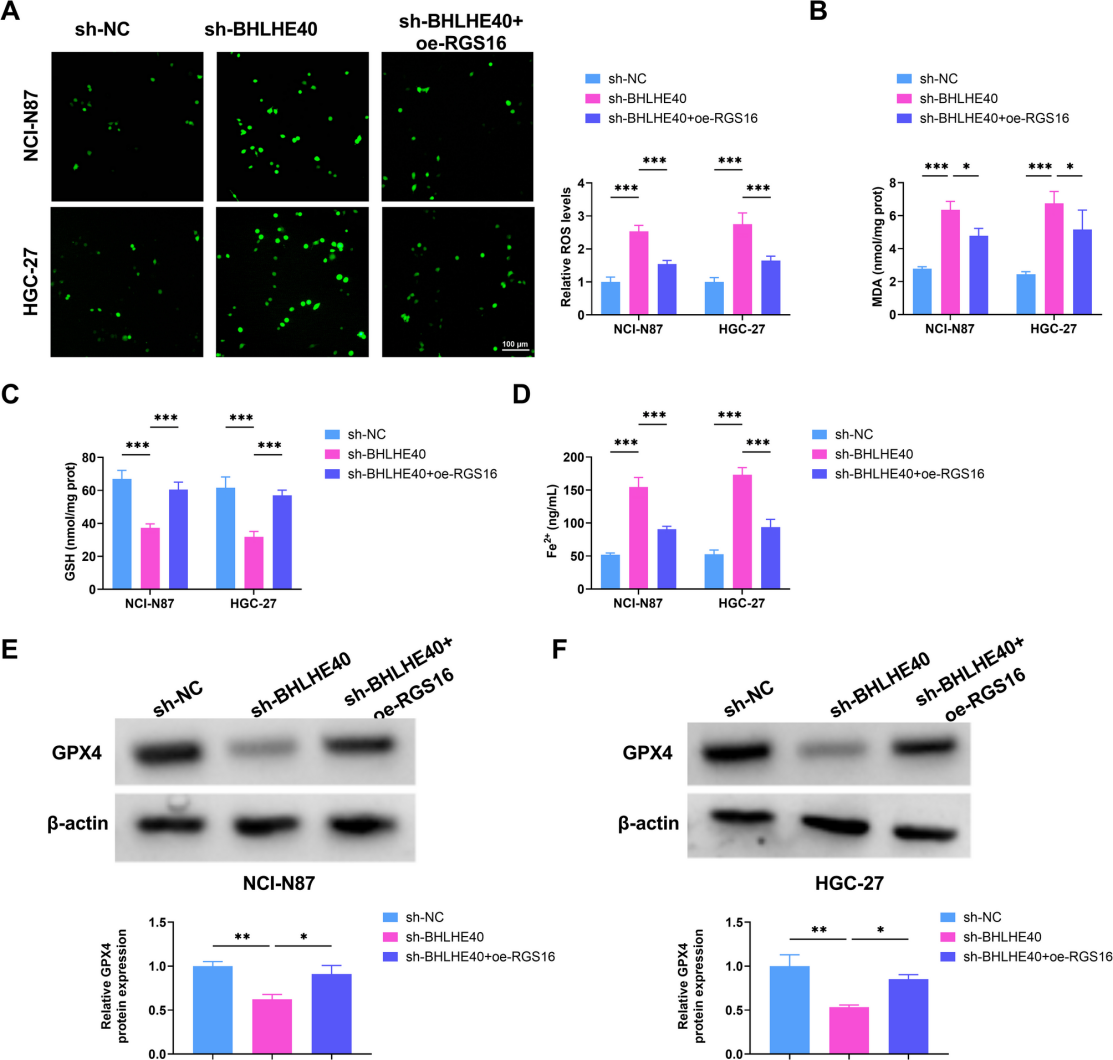
oe-RGS16 reverses the effect of sh-BHLHE40 on ferroptosis and oxidative stress of GC cells. (**A**) Observation of ROS levels in GC cells by a fluorescence microscope. (**B**-**D**) Measurement of MDA, GSH, and Fe^2+^ levels in GC cell lines using corresponding kits. (**E**-**F**) GPX4 protein levels in NCI-N87 and HGC-27 cells transfected with sh-NC, sh-BHLHE40, and sh-BHLHE40 + oe-RGS16 were tested using Western blot. * *P* < 0.05, ** *P* < 0.01, *** *P* < 0.001

The levels of ROS, MDA, GSH, and Fe^2+^ were measured using corresponding kits. BHLHE40 knockdown led to elevated ROS, MDA and Fe²⁺ levels and a diminished GSH level. These changes were all annulled by RGS16 overexpression (Fig. 7A-D). Furthermore, Western blot analysis demonstrated that sh-BHLHE40 considerably hampered the expression of GPX4 protein. However, this dampening effect could be alleviated by the overexpression of RGS16 (Fig. 7E-F). As a result, the exacerbation of ferroptosis and oxidative stress brought by BHLHE40 silencing was counteracted by the upregulation of RGS16.

## Discussion

GC is a highly prevalent and lethal malignant tumor globally, menacing human health severely [32]. Currently, the treatment of GC encompasses multiple modalities such as surgery, chemotherapy, radiotherapy, and emerging targeted and immunotherapies [33, 34]. However, the prognosis of patients is still far from satisfactory, the challenges of recurrence and metastasis urgently need to be overcome, and the exploration of individualized and precise treatment plans still has a long way to go [35, 36]. Thus, an urgent need exists for effective GC therapies to improve the survival.

It has been reported that RGS16 correlates with the malignant progression of various cancers [37–40]. A previous research has confirmed that RGS16 curbs breast cancer cell growth by regulating the Phosphatidylinositol 3-Kinase (PIK3) signaling pathway [41]. Zhang et al. demonstrated that RGS16 could boost YAP activity by interfering with the interaction between LATS1 and MST1, thereby facilitating the proliferation and migration of esophageal cancer cells (ESCC) [25]. Besides, numerous studies have shown that RGS16 can act as a biomarker for cancer diagnosis and prognosis [37, 42, 43]. Nevertheless, the function of RGS16 in GC cells is much less explored. In the current study, RGS16 was proved to be upregulated in GC. When RGS16 was knocked down, it was found that the malignant behaviors of GC cells were all restrained, indicating that RGS16 contributed to the malignant proliferation and metastasis of GC cells.

As a distinct form of cell death arising in a redox setting, ferroptosis has emerged as a popular research subject in recent years owing to its potential anti-tumor capabilities [44]. In light of this research trend, our study also measured the impact of RGS16 on ferroptosis in GC cells. Strikingly, it was illustrated that the knockdown of RGS16 reinforced ferroptosis of GC cells, which implied that RGS16 played a role in promoting tumor progression by inhibiting ferroptosis. Similarly, the tumor-promoting role of RGS16 was substantiated in vivo. The IHC staining results revealed that the Ki67 level, a protein associated with tumor proliferation, was reduced following the knockdown of RGS16. Meanwhile, the level of GPX4, a protein implicated in ferroptosis, was elevated.

The transcription factor BHLHE40, also known as DEC1, belongs to the bHLH family. It is closely associated with tumor cell proliferation and differentiation, lymphocyte maturation, biological rhythm regulation, immune response, and stress response [45–47]. Research has indicated that BHLHE40 participates in the onset and development of various cancers [48, 49]. In tumor cells, BHLHE40 may affect cell metabolism and proliferation by regulating the expression of certain genes [50]. Meanwhile, the GPCR signaling pathway in which RGS16 is involved is also closely related to cell metabolism and proliferation [23, 51]. Therefore, there may be potential interactions between them in these processes. Given the significant role of RGS16 in GC cells, we next sought to explore its upstream regulatory factors, leading us to BHLHE40. The possible transcription factors of RGS16 in GC were predicted through the hTFtarget website, and three transcription factors (BRD3, CTCF, and BHLHE40) were screened out. After knockdown experiments were conducted, it was found that the impact of BHLHE40 knockdown on the expression of RGS16 mRNA was the most significant. The Jasper website was utilized to predict the binding sites of the two, and it was shown by the results from the TIMER database that a positive correlation existed between their expression levels. Subsequent empirical experiments further confirmed that BHLHE40 could be bound to the promoter of RGS16. It has been demonstrated by a previous study that BHLHE40 is upregulated in GC [52]. In parallel with this research, our research likewise verified that BHLHE40 had a high expression level in GC cells. Recent investigations have implicated the crucial role of BHLHE40 in GC. One research discovered that knocking down BHLHE40 could retard the growth, mobility, and glycolysis of GC cells. This was achieved by inhibiting GRIN2D transcription, indicating BHLHE40’s regulatory function in cell behaviors and metabolic processes [53]. Another study showed that the elevated expression of BHLHE40 played a vital part in hypoxia regulation and cell proliferation within GC. Collectively, these findings spotlight BHLHE40 as a key factor in GC development [54]. We thus hypothesized that BHLHE40 might fuel GC invasion by regulating RGS16 expression. This speculation was corroborated by the finding that RGS16 expression was downregulated upon BHLHE40 silencing in GC cells. Concurrently, the silencing of BHLHE40 notably curbed the malignant behaviors of GC cells, while these inhibitory outcomes were overturned by the upregulation of RGS16. Similarly, the effect of facilitating ferroptosis in GC cells by knocking down BHLHE40 could be reversed with the overexpression of RGS16. Hence, it can be inferred that BHLHE40 impacts the progression of GC via modulating RGS16 expression. These results suggest that RGS16 holds potential therapeutic significance for combating GC.

Since our study utilized open data from GEO, it is crucial to reference TCGA, the most widely used open cancer dataset. Many prior studies have leveraged TCGA data to identify potential cancer biomarkers. Researchers have explored the diagnostic, prognostic, and therapeutic values of various genes across different cancer types using TCGA data [55, 56]. Additionally, Liu’s Lab, one of the earliest groups working on TCGA biomarker studies, has made significant contributions by developing diverse strategies for TCGA-based analyses. Their works have provided valuable insights into cancer biomarker discovery and genetic mechanisms [18, 57]. However, although these studies demonstrate substantial productivity, their high-volume publication in batches should be addressed critically. We need to evaluate whether this approach impacts research depth or introduces methodological redundancies. For example, some studies might focus too much on data mining and overlook in-depth experimental validation, or there could be overlaps in the analytical methods used across different studies. Furthermore, the limitations of TCGA data should also be considered. TCGA data has several advantages, such as providing a large amount of multi-omics data from a wide range of cancer patients, which is useful for large-scale biomarker discovery and understanding the molecular mechanisms of cancer. However, it also has potential biases. Technical biases may arise from differences in sequencing platforms, sample preparation methods, and data processing pipelines. Biological biases can be caused by factors such as tumor heterogeneity, differences in patient populations, and the presence of contaminants in the samples. Sample selection biases may lead to non-representative data, affecting the generalizability of the research findings. A balanced discussion of these aspects is essential to ensure the accurate interpretation of TCGA data in our study.

Recent studies have highlighted significant advancements in liquid biopsies for cancer diagnostics and monitoring [58]. Emerging sequencing technologies have enhanced the sensitivity and specificity of DNA analysis [59]. Moreover, DNA methylation has also been explored as a biomarker for early cancer detection [60]. In the context of our study, it is worth considering whether the mechanisms we analyzed, such as the role of BHLHE40 and RGS16 in GC, could be identified using these diagnostic methods. For example, ctDNA analysis might be able to detect changes in the expression or methylation status of BHLHE40 and RGS16 genes in the circulation, providing a non-invasive way to monitor the disease progression. However, further research is needed to validate the feasibility and accuracy of using liquid biopsy techniques in our specific research context.

Future studies should aim to validate these findings in patient-derived xenograft (PDX) models. Previous research utilizing xenograft models for cancer investigation, provides a basis for our future research [61–63]. Xenograft models have several advantages in studying cancer mechanisms. They can better mimic the in vivo tumor microenvironment compared to cell culture models, allowing for more accurate evaluation of the biological functions of genes and the effects of therapeutic interventions. However, they also have limitations. For example, there may be differences in the growth and behavior of tumors in xenograft models compared to human tumors due to species differences. The immune system of the host animals may also affect the growth and development of the xenografted tumors. In the context of our study, using PDX models to validate the role of BHLHE40 and RGS16 in GC progression will help to further confirm our findings and provide more reliable evidence for potential therapeutic strategies.

On the whole, our research findings spotlight that RGS16 functions as an oncogenic role in GC. Moreover, the upregulation of RGS16, which is mediated by the transcription factor BHLHE40, can boost the progression of GC. This discovery not only enriches our comprehension of GC’s molecular mechanisms but also heralds potential therapeutic targets. Moving forward, efforts should center on devising strategies to disrupt their axis, aiming to reactivate ferroptosis and impede GC advancement, thus holding great promise for enhancing patient outcomes in the battle against this lethal disease.

## Electronic supplementary material

Below is the link to the electronic supplementary material.

Supplementary Material 1

Supplementary Material 2

## Data Availability

No datasets were generated or analysed during the current study.
