# Supplementary material for: BHLHE40-mediated RGS16 upregulation: a driver propelling gastric cancer progression via ferroptosis suppression
Source: Hereditas. 2025 May 24;162:87. doi: 10.1186/s41065-025-00447-y (PMC12102885; doi:10.1186/s41065-025-00447-y)
Supplement: Supplementary file 1 — Supplementary Material 1 [file 41065_2025_447_MOESM1_ESM.pdf]

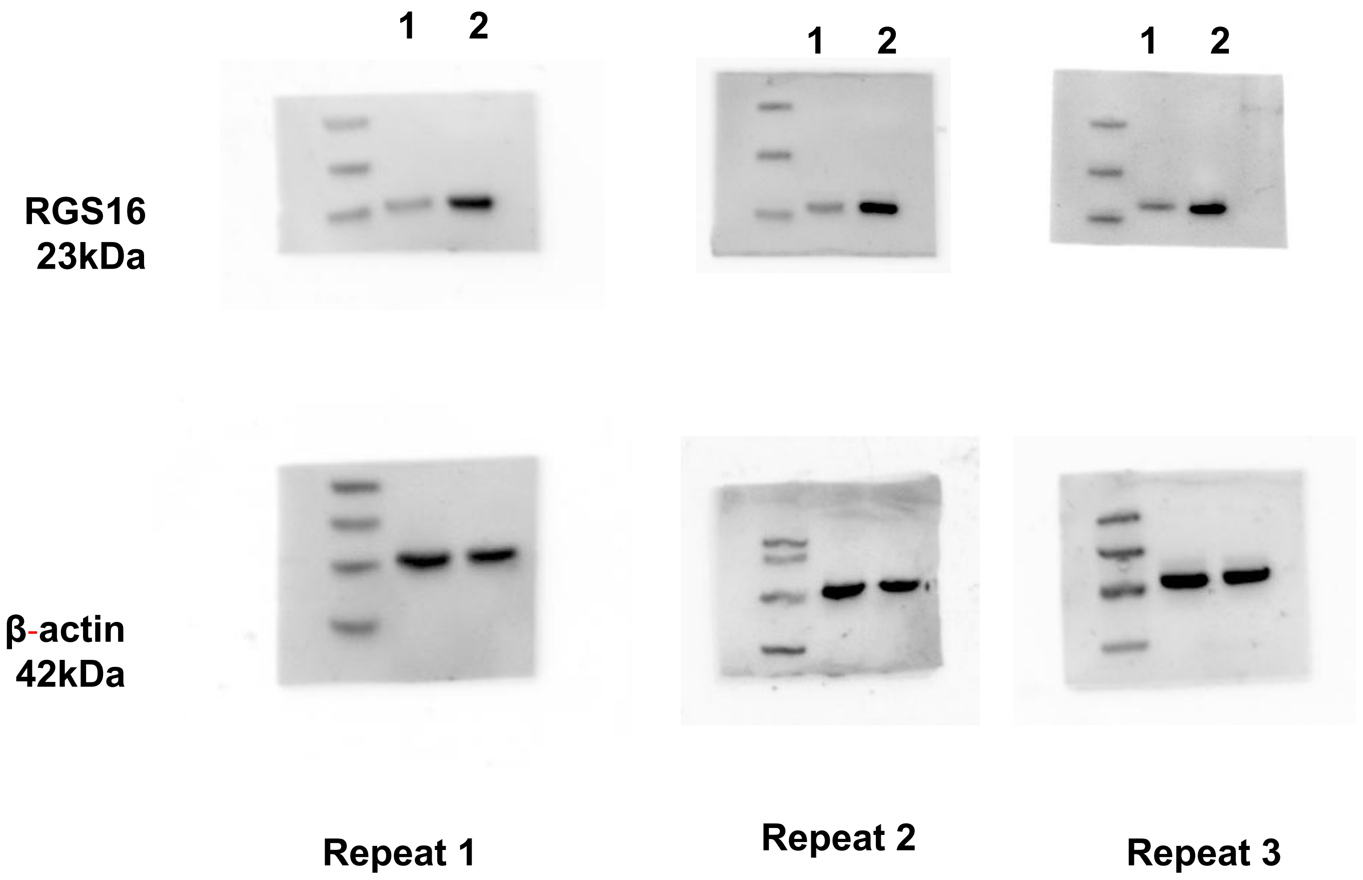

Fig1e

1 Normal

2 Tumor

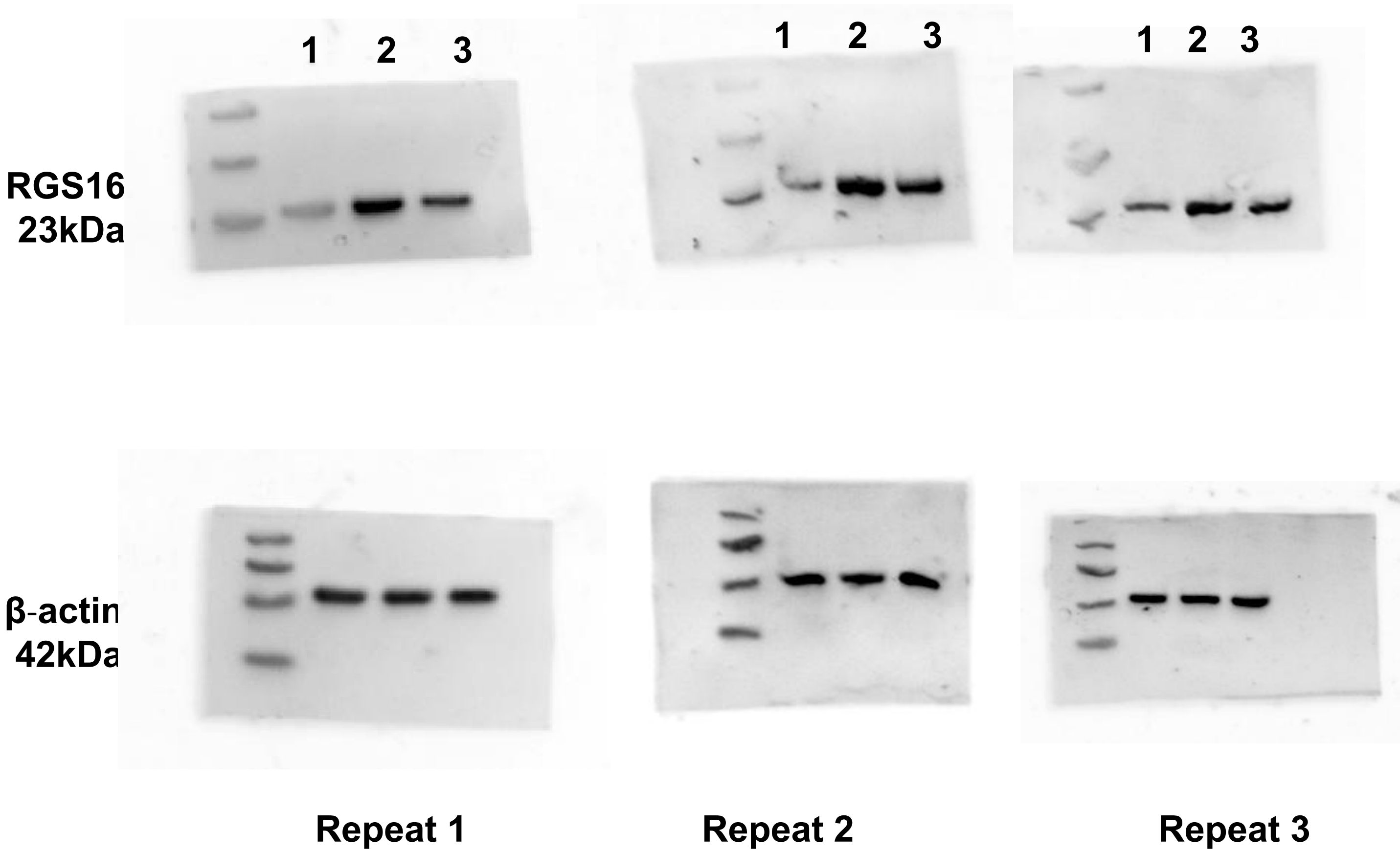

Fig1G 1 GSE-1

2 NCI-N87

3 HGC-27

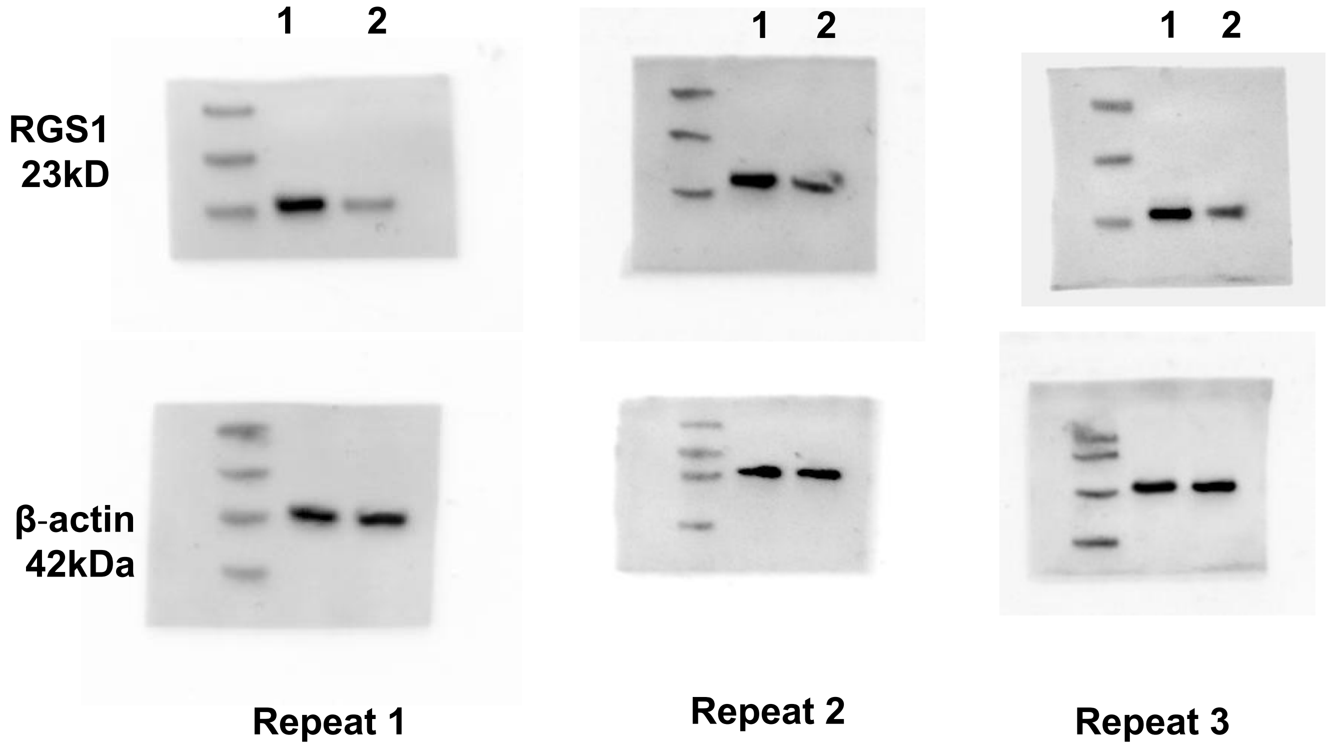

Fig 2A

1 sh-NC

2 sh-RGS16

NCI-N87

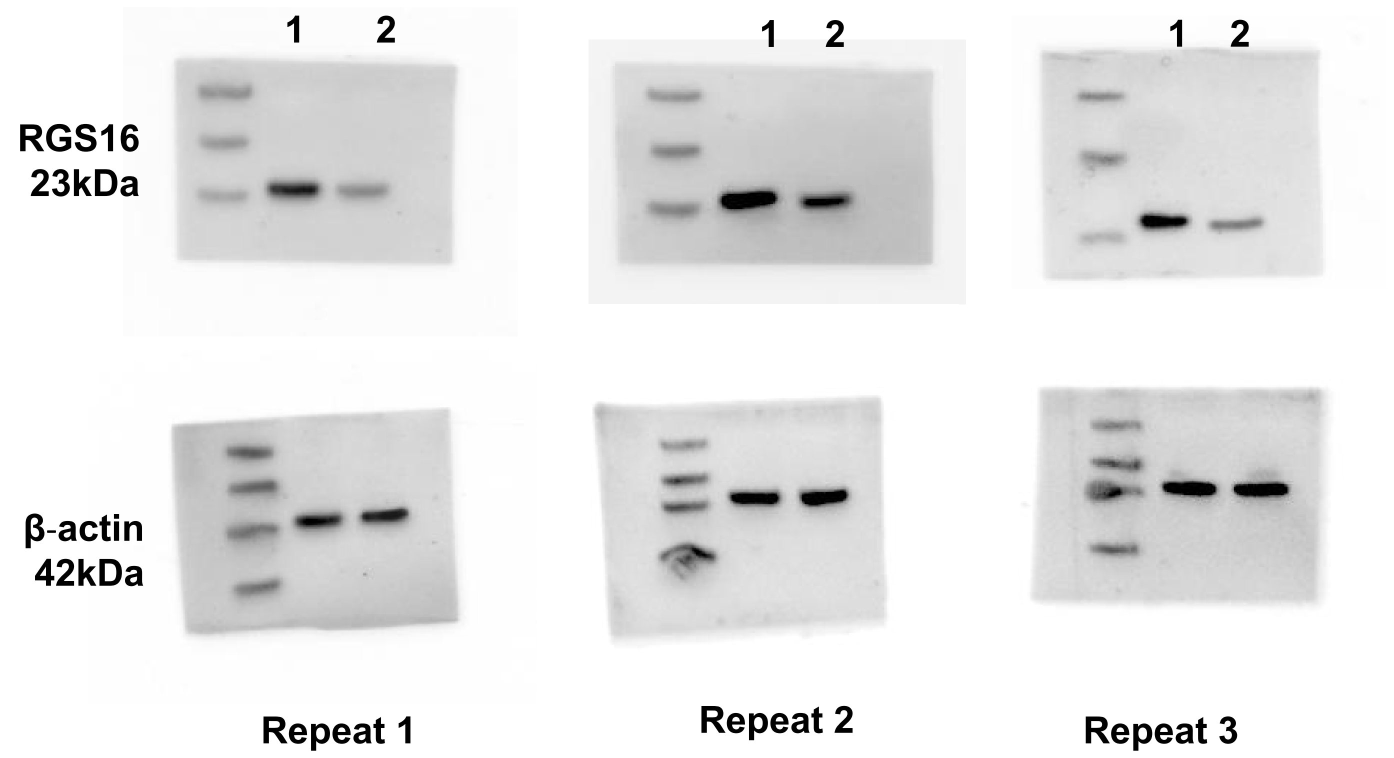

Fig 2A

1 sh-NC

2 sh-RGS16

HGC -27

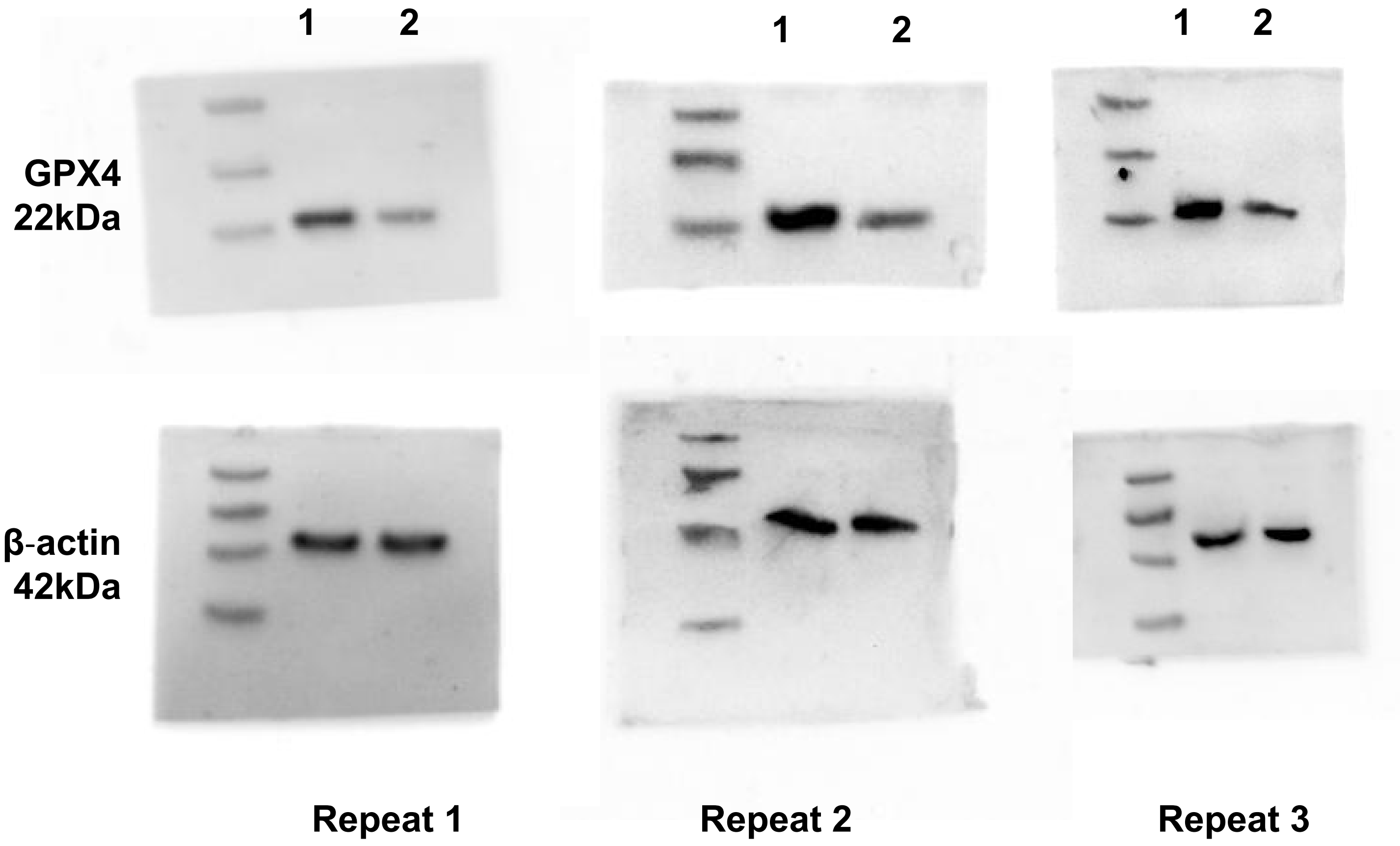

Fig3E 1 sh-NC

2 sh-RGS16

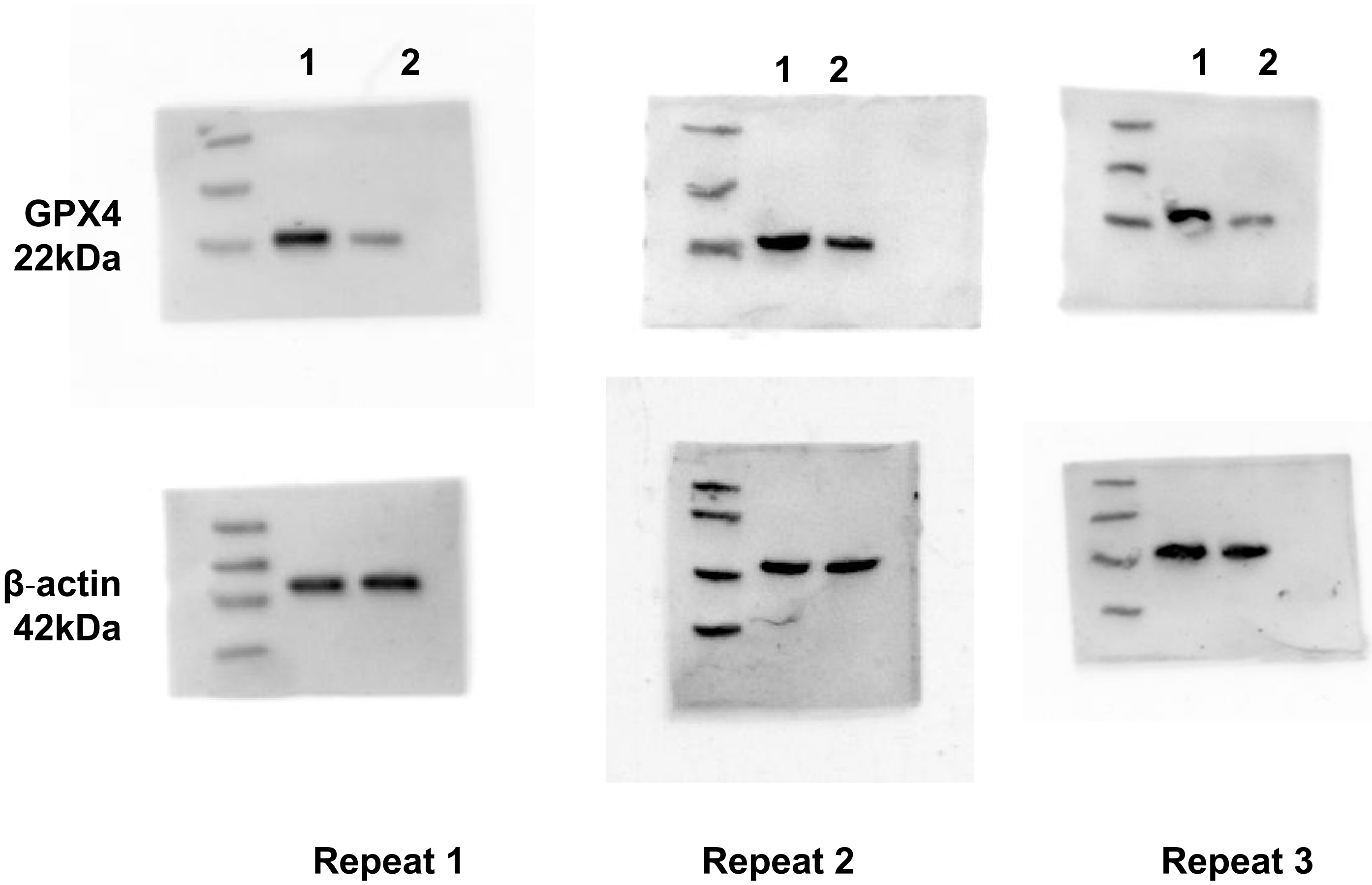

Fig3F 1 sh-NC

2 sh-RGS16

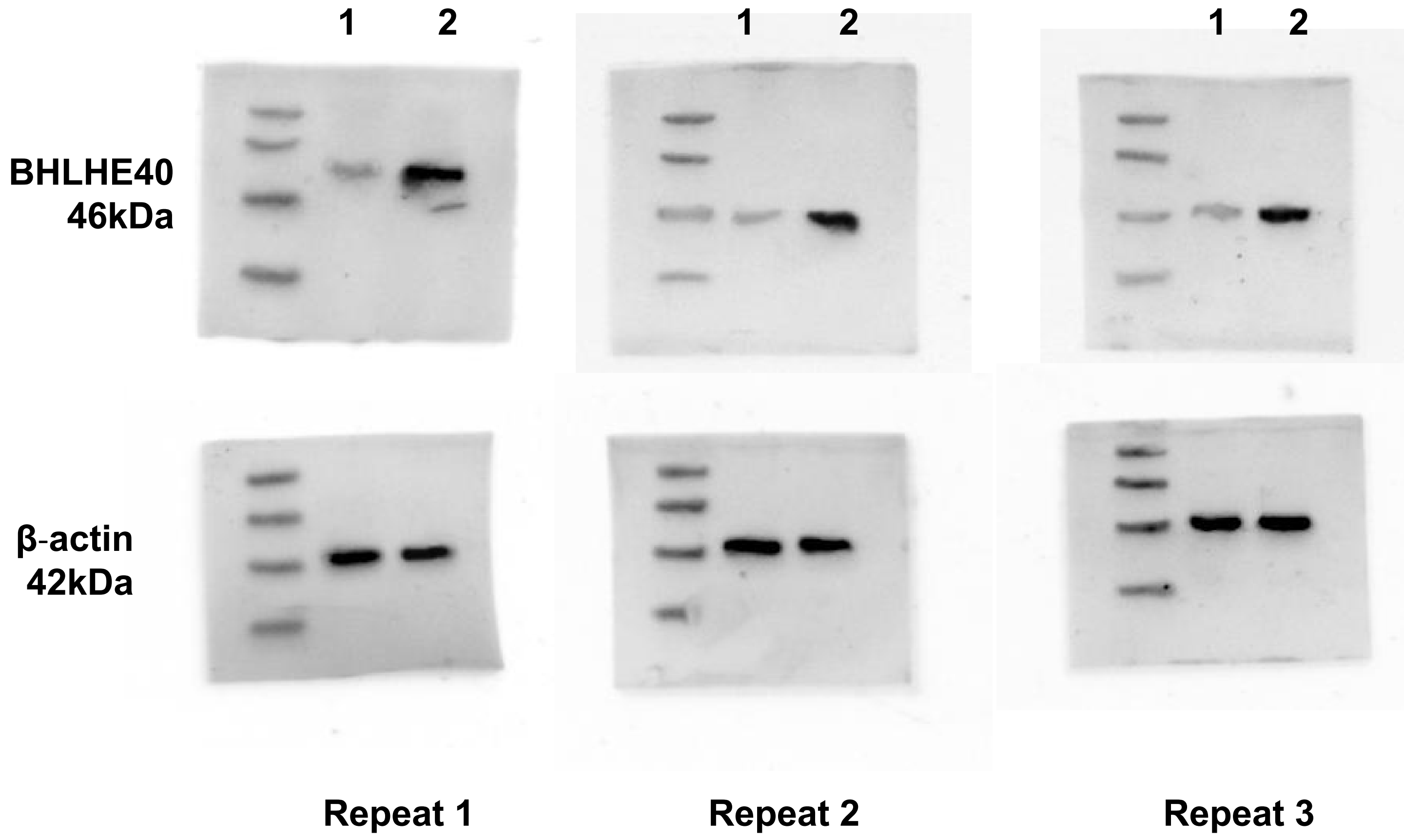

Fig5H

1 Normal

2Tumor

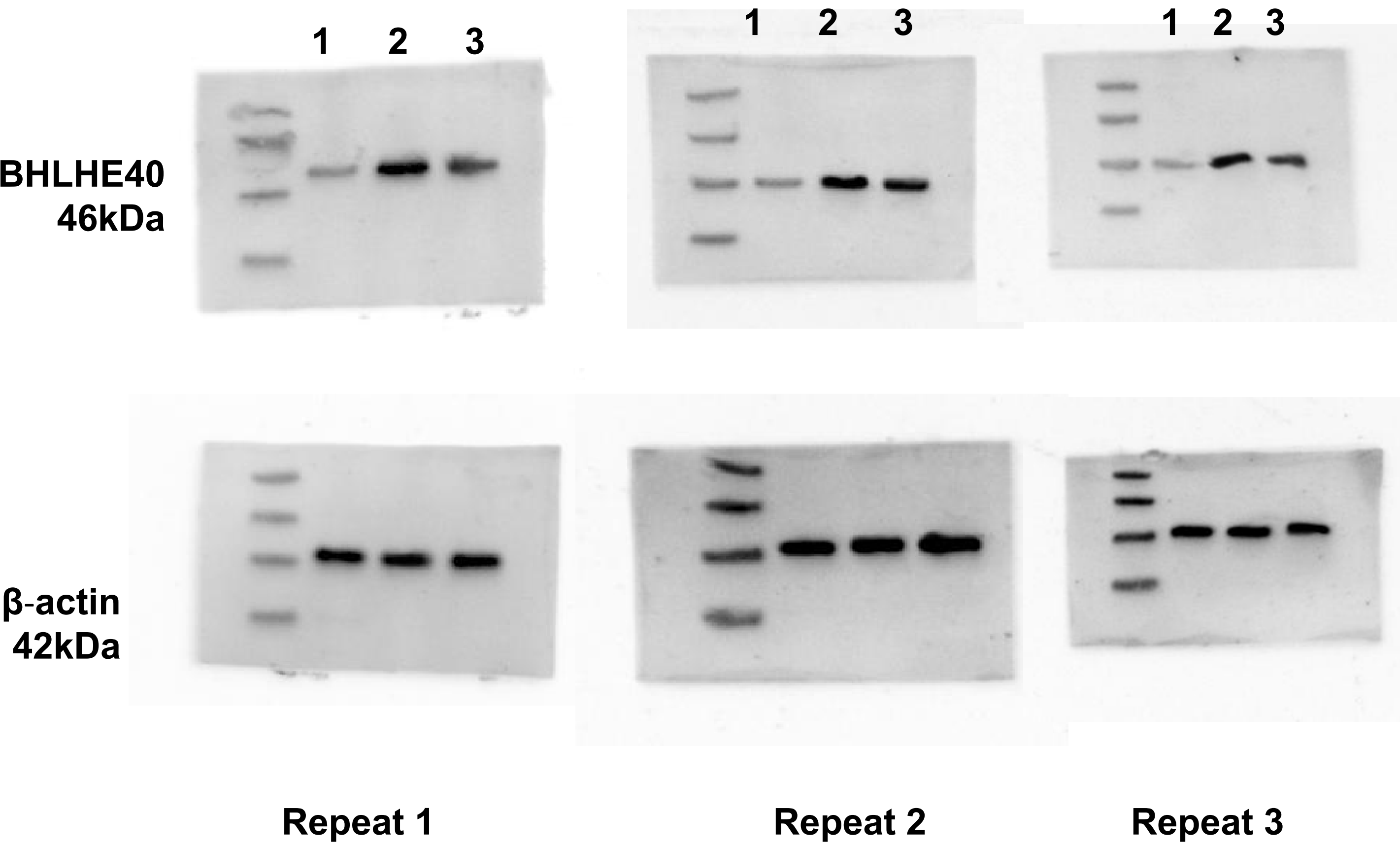

Fig5J

1 GSE-1

2 NCI-N87

3 HGC-27

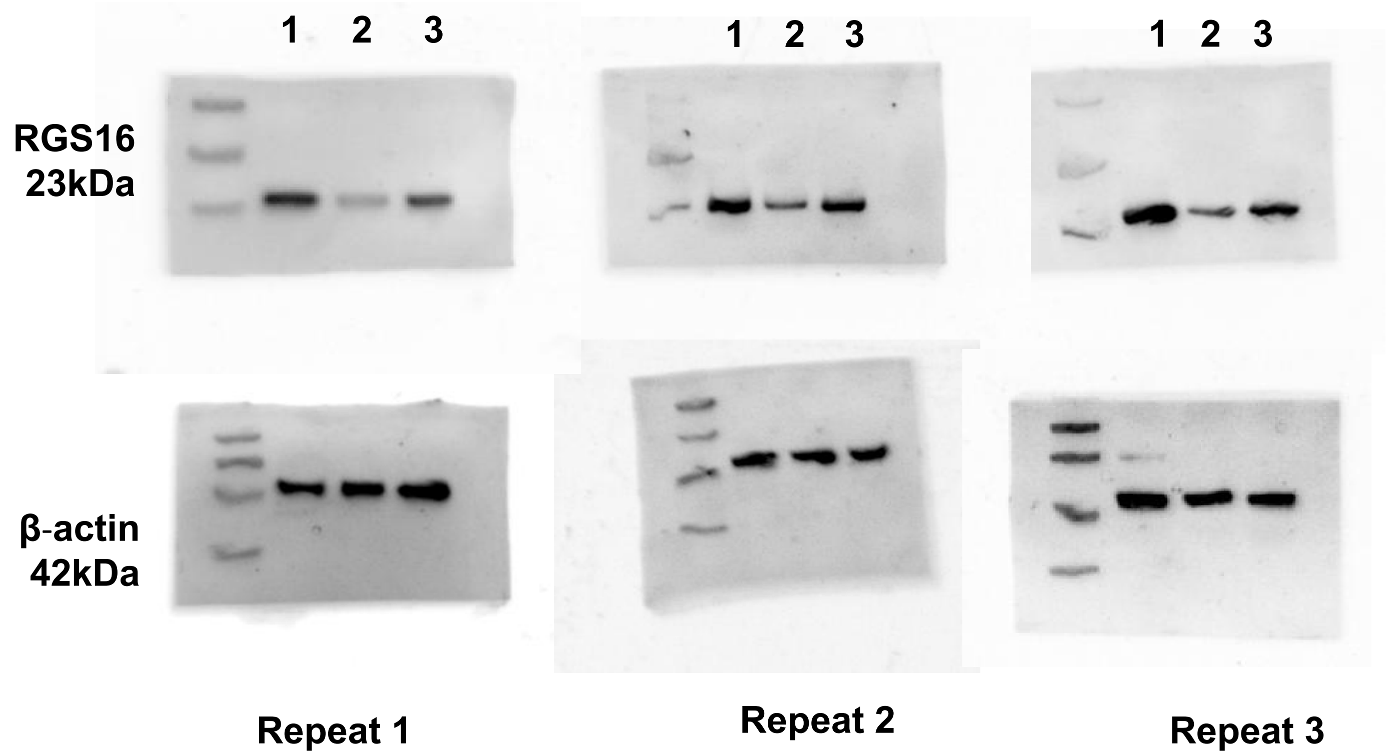

Fig6A

1 sh-NC

2 sh-BHLHE40

2 sh-BHLHE40+oe-RGS16

NCI-N87

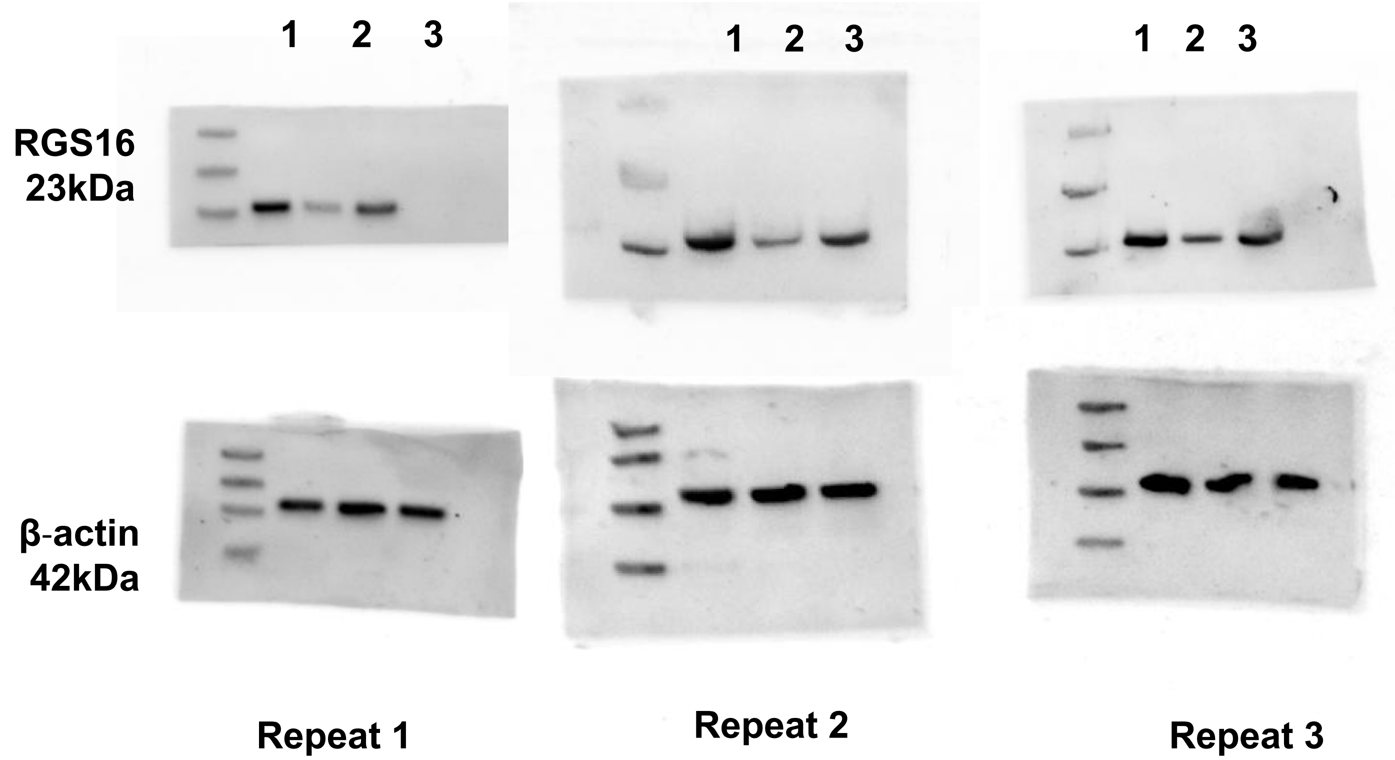

Fig6A

1 sh-NC

2 sh-BHLHE40

3 sh-BHLHE40+oe-RGS16

HGC -27

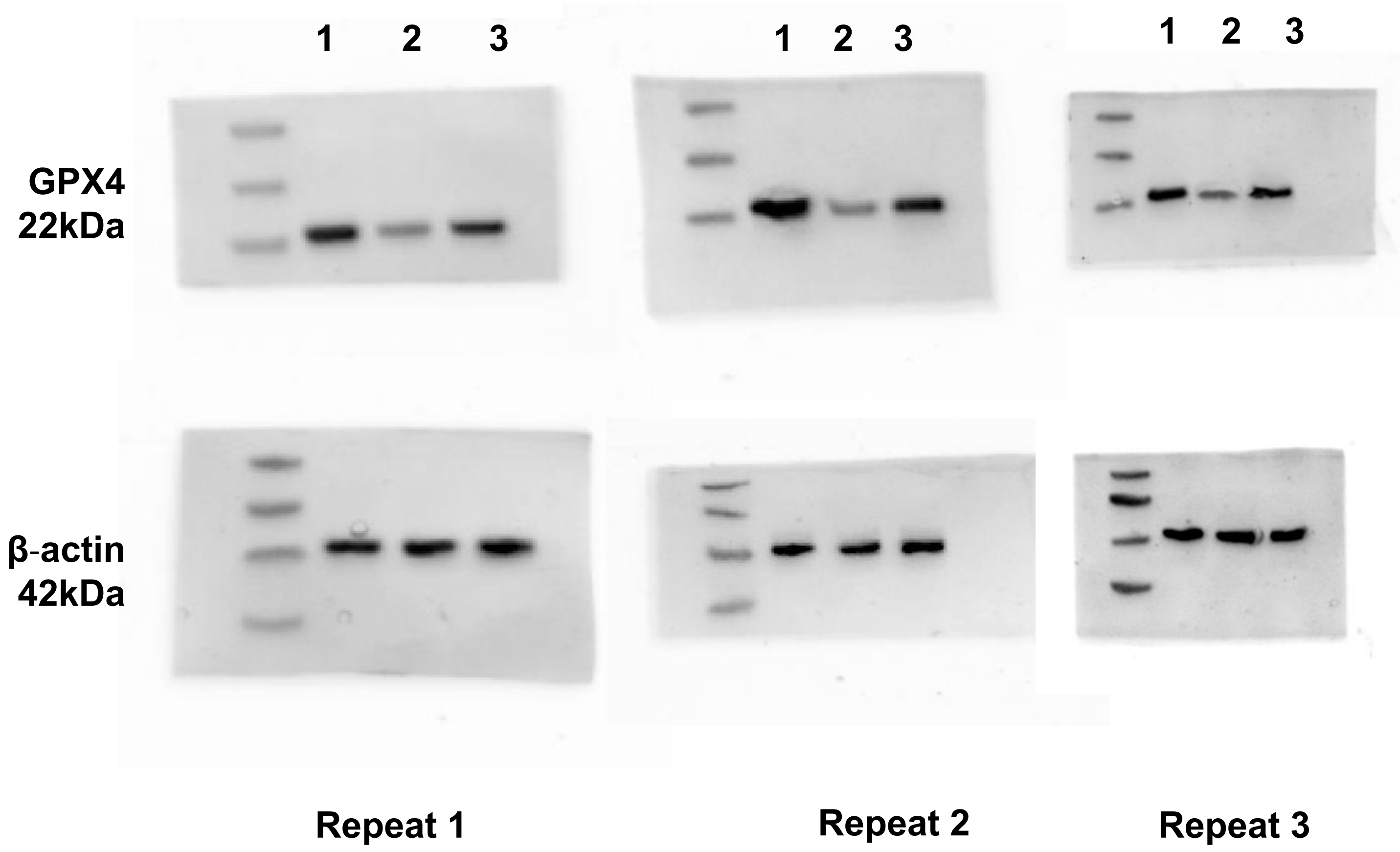

Fig7E 1 sh-NC

2 sh-BHLHE40

3 sh-BHLHE40+oe-RGS16

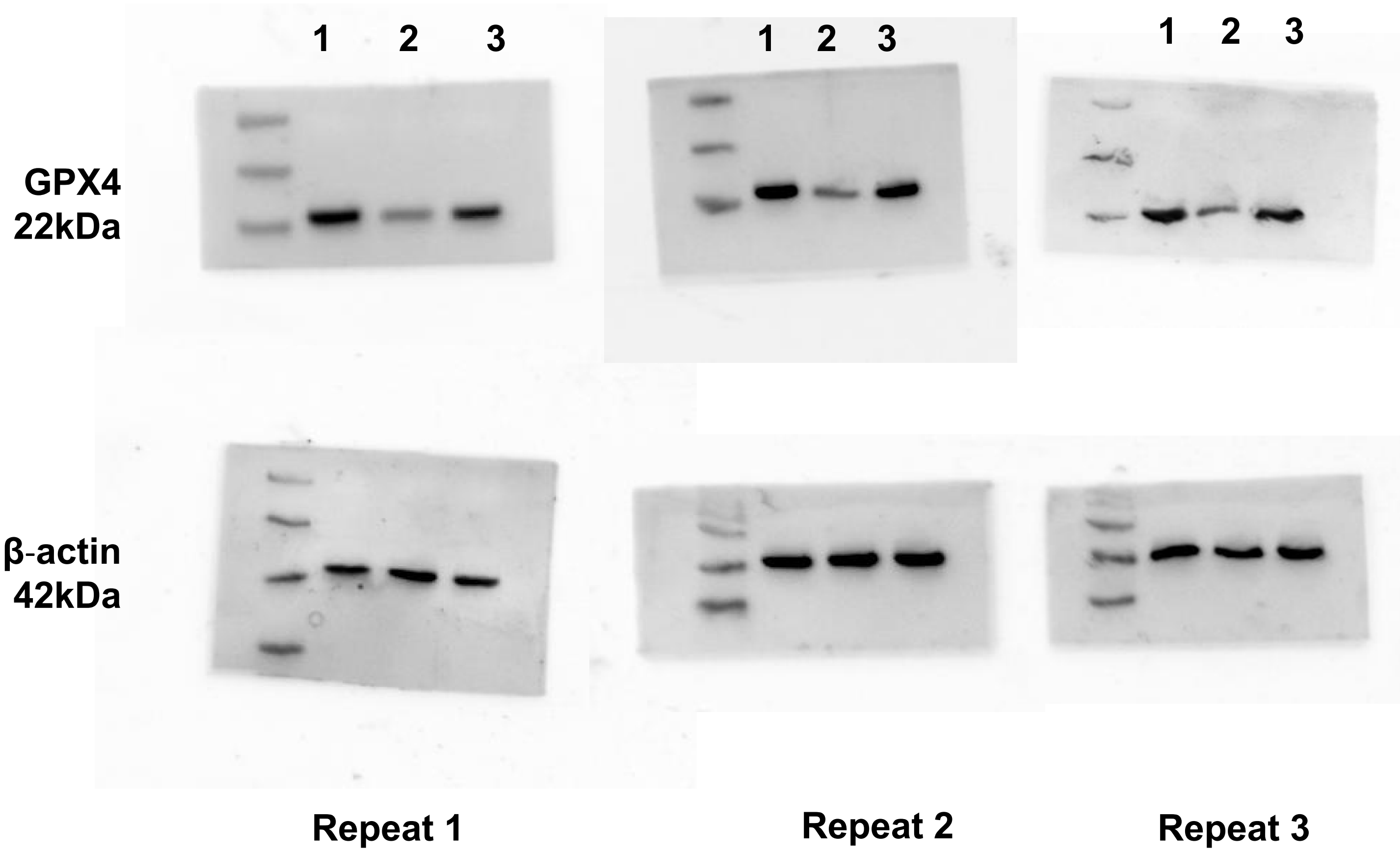

Fig7F 1 sh-NC

2 sh-BHLHE40

3 sh-BHLHE40+oe-RGS16

Protein Marker

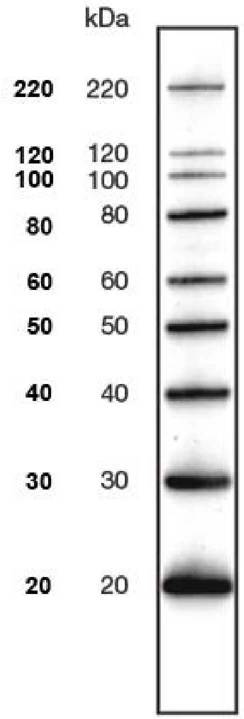

- **The samples derive from the same experiment and that gels/blots were processed in parallel.**
